# Supplementary material for: A genome-wide relay of signalling-responsive enhancers drives hematopoietic specification
Source: Nat Commun. 2023 Jan 17;14:267. doi: 10.1038/s41467-023-35910-9 (PMC9845378; doi:10.1038/s41467-023-35910-9)
Supplement: Supplementary file 1 — Supplementary Information [file 41467_2023_35910_MOESM1_ESM.pdf]

A genome-wide relay of signalling-responsive enhancers drives hematopoietic specification

Edginton-White, B.[1+*], Maytum, A[1+]; Kellaway, S.G.[1], Goode, D.K.[2], Keane, P.[1], Pagnuco, I.[3,1], Assi, S.A.[1], Ames, L.[1], Clarke, M[1], Cockerill, P.N., Göttgens, B.[2], Cazier, J.B.[1,3], and Bonifer, C[1*].

Supplementary Information

1. Supplementary Figures
2. Supplementary Notes

1. Supplementary Figures

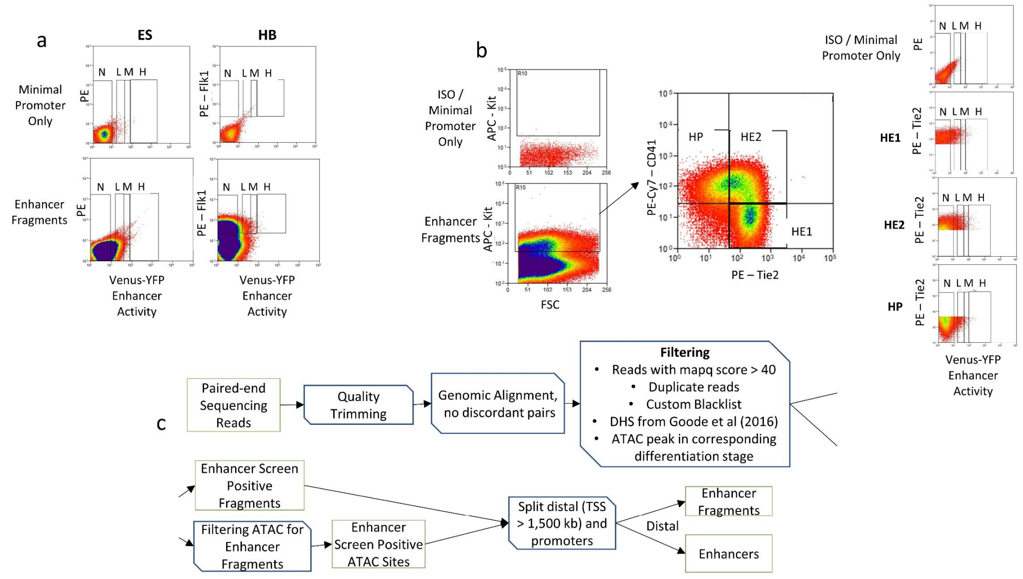

d

Overlap of plasmid libraries with ATAC-Seq Peaks

	ATAC-Seq Peaks		pDONR		pSKB	
	Promoter	Distal	Promoter	Distal	Promoter	Distal
**ES**	14184	35358	98.4%	85.1%	98.0%	80.8%
**HB**	14449	32159	99.1%	96.8%	98.9%	92.9%
**HE1**	14070	34351	98.4%	97.4%	97.6%	89.5%
**HE2**	14259	34784	98.3%	96.8%	97.0%	84.4%
**HP**	14125	33878	99.2%	98.3%	97.6%	84.1%

e

	Positive Fragments	Distal	Promoter	Distal %	Promoter %
**ES**	125,020	27,717	97,303	22.2	77.8
**HB**	153,764	38,953	114,811	25.3	74.7
**HE1**	326,645	101,943	224,702	31.2	68.8
**HE2**	219,410	68,427	150,983	31.2	68.8
**HP**	144,911	42,484	102,427	29.3	70.7

	Negative Fragments	Distal	Promoter	Distal %	Promoter %
**ES**	47,862	10,352	37,510	21.6	78.4
**HB**	34,991	8,426	26,565	24.1	75.9
**HE1**	134,849	41,374	93,475	30.7	69.3
**HE2**	26,754	8,430	18,324	31.5	68.5
**HP**	31,331	9,017	22,314	28.8	71.2

f

Number of Enhancer Positive Distal ATAC Sites

	Rep 1	Rep 2	Overlap	Total
ES	9,968	948	760	10,156
HB	11,884	1,334	1,156	12,062
HE1	18,109	2,148	2,077	18,180
HE2	16,868	470	447	16,891
HP	13,267	579	523	13,323

Number of Positive Promoter ATAC sites

	Rep 1	Rep 2	Overlap	Total
ES	11,407	2,679	2,642	11,444
HB	11,810	2,983	2,958	11,835
HE1	12,225	4,938	4,923	12,240
HE2	12,077	1,396	1,386	12,087
HP	11,621	1,486	1,474	11,633

g

	Median number of unique positive fragments per distal ATAC site
**ES**	3
**HB**	4
**HE1**	6
**HE2**	4
**HP**	3

Supplementary Fig. 1: Number of stage specific enhancer and promoter fragments scoring positive in the assay. a,b) Sorting and analysis of cells with activated reporter construct, a) example of detection of enhancer activity as compared to the minimal promoter for ESCs and HB, b) representative sorting picture for all differentiation stages used for all FACS experiments and gating strategy for enhancer reporter library (right panel), c) Outline of the filtering pipeline, d) Overlap of the coverage of ATAC-Seq sites in the two plasmid libraries pDONR and pSKB for the different differentiation stages; e) total number of distal and promoter fragments scoring either positive or negative recovered in the assay, f) Number of distal and promoter ATAC-sites recovered in two replicates of the assay (for more details see Supplementary Notes). (g) Median number of enhancer positive fragments per ATAC site.

a

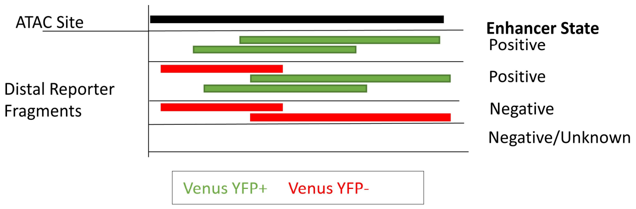

b

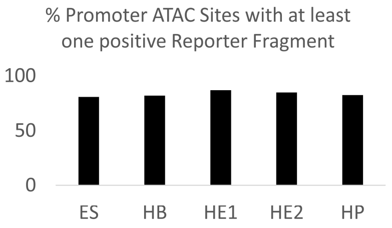

c

	H3K27Ac ChIP Goode et al 2016	Enh Positive ATAC Sites	Overlap	% Enh overlapping with H3K27Ac ChIP
**ES**	108,485	9,968	2,954	29.63
**HB**	72,240	11,884	5,858	49.29
**HE1**	53,160	18,109	11,948	65.98
**HE2**	53,160	16,868	10,671	63.26
**HP**	97,254	13,267	5,965	44.96

d

Current VISTA database: 1061 enhancers

	No. of Enhancer Positive ATAC sites found in VISTA	No. of Enhancer Negative/Unknown ATAC sites found in VISTA
ES	189	118
HB	239	157
HE1	321	90
HE2	301	98
HP	240	118

e

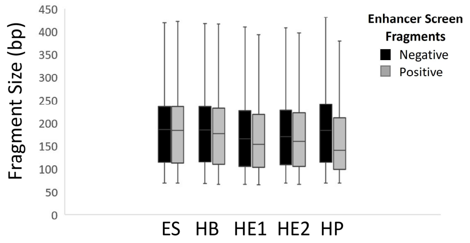

f

E9.5 ATAC Sites (Howell, et al. 2021) : 99,297

	No. of Enhancer Positive ATAC sites Overlapping E9.5	Percentage of Enhancer Positive ATAC sites found in E9.5
**ES**	7,956	79.8%
**HB**	10,088	84.9%
**HE1**	16,030	88.5%
**HE2**	14,749	87.4%
**HP**	11,345	85.5%

E13.5 ATAC Sites (Howell, et al. 2021) : 96,536

	No. of Enhancer Positive ATAC sites Overlapping E13.5	Percentage of Enhancer Positive ATAC sites found in E13.5
**ES**	7,494	75.2%
**HB**	9,453	79.5%
**HE1**	14,918	82.4%
**HE2**	13,906	82.4%
**HP**	10,895	82.1%

g

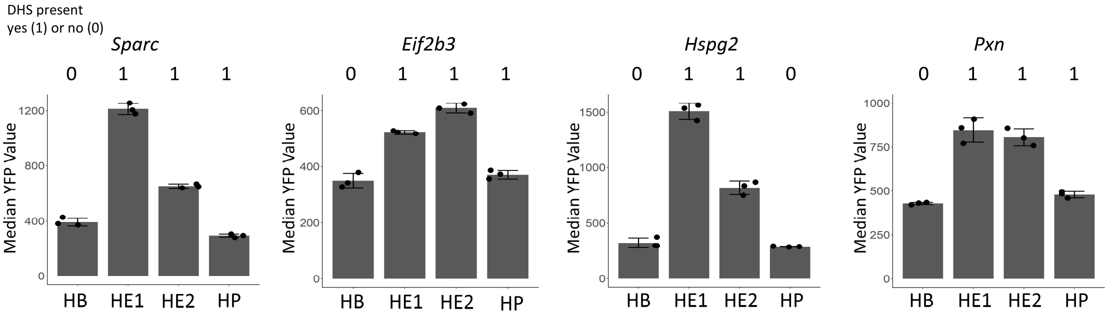

Supplementary Fig. 2: Enhancer characteristics. a) Example of a hypothetical ATAC site showing 4 possible outcomes for the specification of different types of open chromatin fragments and their correlation with the YFP signal. Fragments are labelled as scoring negative if they do not stimulate reporter gene activity, fragments are labelled as unknown if they are present in the original ATAC-Seq library but are neither present in the positive nor the negative fraction. b) Percentage of promoter ATAC sites containing at least one fragment scoring positive in our assay Source data are provided as a Source Data file. c) Number of distal fragments scoring positive in our assay overlapping with H3K27Ac marked chromatin

sites (Data from Goode at el., 2016)[1]. d) Number of distal fragments scoring positive in our assay found in the VISTA database; e) size of positive and negative distal fragments detected in the assay. N=between 8,430 and 101,943 distal enhancer screen fragments (see Supplementary Fig. 1e for individual numbers) (line shows median value, boxes show the interquartile ranges and the whiskers indicate 1.5 x interquartile range, no outliers shown) Source data are provided as a Source Data file. f) overlap of distal fragments scoring positive in our assay with open chromatin sites in the hemogenic endothelium of mouse embryos (data from [2]). g) Examples of individually validated enhancer elements from left to right. *Sparc* enhancer (Chr11:55405590-55406032), *Eif2b3* enhancer (Chr4: 117056629-117057073), *Hspg2* enhancer element (Chr4:137478251-137478690), *Pxn* enhancer element (Chr5:115530006-115530483). Data are presented as mean values +/-SD. Dots showing individual values for n=3 biologically independent experiments. Source data are provided as a Source Data file. Binary code represents if a DHS is present (1) or not present (0) in each cell type.

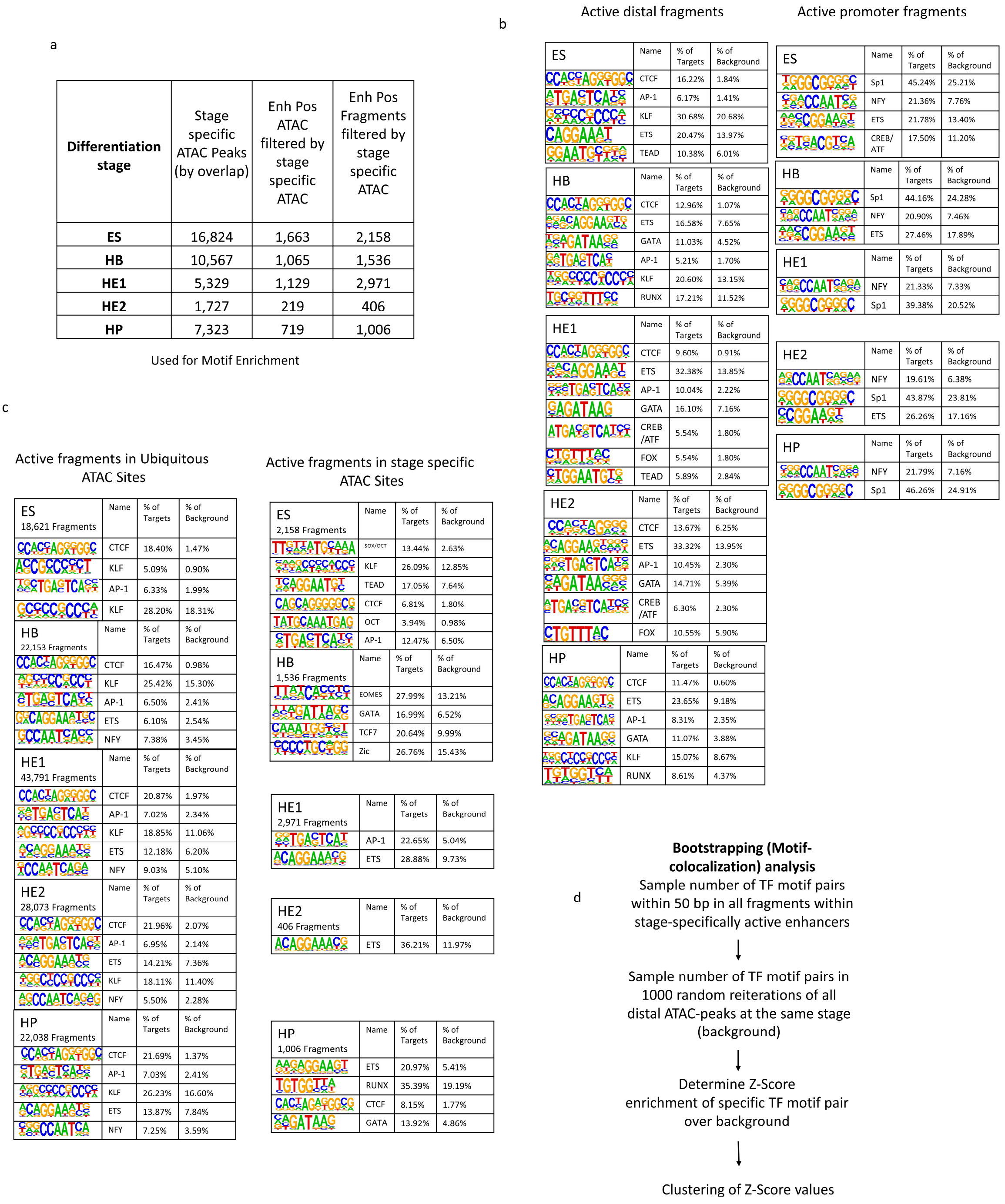

a

**Differentiation stage**	Stage specific ATAC Peaks (by overlap)	Enh Pos ATAC filtered by stage specific ATAC	Enh Pos Fragments filtered by stage specific ATAC
**ES**	16,824	1,663	2,158
**HB**	10,567	1,065	1,536
**HE1**	5,329	1,129	2,971
**HE2**	1,727	219	406
**HP**	7,323	719	1,006

Used for Motif Enrichment

b

Active distal fragments

Stage	Name	% of Targets	% of Background
ES	CTCF	16.22%	1.84%
	AP-1	6.17%	1.41%
	KLF	30.68%	20.68%
	ETS	20.47%	13.97%
	TEAD	10.38%	6.01%
HB	CTCF	12.96%	1.07%
	ETS	16.58%	7.65%
	GATA	11.03%	4.52%
	AP-1	5.21%	1.70%
	KLF	20.60%	13.15%
	RUNX	17.21%	11.52%
HE1	CTCF	9.60%	0.91%
	ETS	32.38%	13.85%
	AP-1	10.04%	2.22%
	GATA	16.10%	7.16%
	CREB /ATF	5.54%	1.80%
	FOX	5.54%	1.80%
	TEAD	5.89%	2.84%
HE2	CTCF	13.67%	6.25%
	ETS	33.32%	13.95%
	AP-1	10.45%	2.30%
	GATA	14.71%	5.39%
	CREB /ATF	6.30%	2.30%
	FOX	10.55%	5.90%
HP	CTCF	11.47%	0.60%
	ETS	23.65%	9.18%
	AP-1	8.31%	2.35%
	GATA	11.07%	3.88%
	KLF	15.07%	8.67%
	RUNX	8.61%	4.37%

Active promoter fragments

Stage	Name	% of Targets	% of Background
ES	Sp1	45.24%	25.21%
	NFY	21.36%	7.76%
	ETS	21.78%	13.40%
	CREB/ ATF	17.50%	11.20%
HB	Sp1	44.16%	24.28%
	NFY	20.90%	7.46%
	ETS	27.46%	17.89%
HE1	NFY	21.33%	7.33%
	Sp1	39.38%	20.52%
HE2	NFY	19.61%	6.38%
	Sp1	43.87%	23.81%
	ETS	26.26%	17.16%
HP	NFY	21.79%	7.16%
	Sp1	46.26%	24.91%

c

Active fragments in Ubiquitous ATAC Sites

Stage	Name	% of Targets	% of Background
ES 18,621 Fragments	CTCF	18.40%	1.47%
	KLF	5.09%	0.90%
	AP-1	6.33%	1.99%
	KLF	28.20%	18.31%
HB 22,153 Fragments	CTCF	16.47%	0.98%
	KLF	25.42%	15.30%
	AP-1	6.50%	2.41%
	ETS	6.10%	2.54%
	NFY	7.38%	3.45%
HE1 43,791 Fragments	CTCF	20.87%	1.97%
	AP-1	7.02%	2.34%
	KLF	18.85%	11.06%
	ETS	12.18%	6.20%
	NFY	9.03%	5.10%
HE2 28,073 Fragments	CTCF	21.96%	2.07%
	AP-1	6.95%	2.14%
	ETS	14.21%	7.36%
	KLF	18.11%	11.40%
	NFY	5.50%	2.28%
HP 22,038 Fragments	CTCF	21.69%	1.37%
	AP-1	7.03%	2.41%
	KLF	26.23%	16.60%
	ETS	13.87%	7.84%
	NFY	7.25%	3.59%

Active fragments in stage specific ATAC Sites

Stage	Name	% of Targets	% of Background
ES 2,158 Fragments	SOX/OCT	13.44%	2.63%
	KLF	26.09%	12.85%
	TEAD	17.05%	7.64%
	CTCF	6.81%	1.80%
	OCT	3.94%	0.98%
	AP-1	12.47%	6.50%
HB 1,536 Fragments	EOMES	27.99%	13.21%
	GATA	16.99%	6.52%
	TCF7	20.64%	9.99%
	Zic	26.76%	15.43%
HE1 2,971 Fragments	AP-1	22.65%	5.04%
	ETS	28.88%	9.73%
HE2 406 Fragments	ETS	36.21%	11.97%
HP 1,006 Fragments	ETS	20.97%	5.41%
	RUNX	35.39%	19.19%
	CTCF	8.15%	1.77%
	GATA	13.92%	4.86%

Supplementary Fig. 3: Dynamics of TF binding motif enrichment in cell type specific accessible chromatin sites, enhancer and promoter elements. a) Number of stage specific ATAC peaks and ATAC fragments with enhancer activity co-localizing with stage specific ATAC sites, b) TF binding motif enrichment analysis of distal and promoter fragments scoring positive in our assay (active) using the HOMER software[3]. c) TF binding motif enrichment analysis of enhancer fragments scoring positive in our assay (active) derived from ubiquitous and stage specific ATAC sites. Position-weight matrices used for this analysis can be found in Supplementary Table 1 in Supplementary Notes. d) Overview over the motif colocalization analysis (Fig. 3).

a

	All	Promoters	Distal
**HB**	74.10	80.96	70.83
**HE1**	78.20	83.49	74.53
**HE2**	63.27	79.07	56.66
**HP**	77.21	90.48	49.65

Percentage of ATAC peaks from serum free culture overlapping with ATAC peaks from serum culture

b

Cytokine	Cell type	#Distal elements	#Distal elements identified as enhancers	**#Two-fold increase enhancers**	**#Two-fold decrease enhancers**	#Promoter elements	**#Two-fold increase promoters**	**#Two-fold decrease promoters**
VEGF	HE1	14739	7197	**2426**	**1357**	14585	**1405**	**353**
	HE2	13964	7720	**1733**	**2008**	15894	**1661**	**311**
	HP	18256	7834	**622**	**1331**	12896	**1204**	**506**
IL6	HE1	25437	9746	**2993**	**1139**	15404	**497**	**248**
	HE2	16991	8275	**303**	**247**	15735	**162**	**55**
	HP	5864	1639	**144**	**253**	9047	**572**	**168**
IL3	HE1	34042	13667	**248**	**283**	15992	**141**	**97**
	HE2	12040	6584	**349**	**334**	15411	**322**	**111**
	HP	35691	10906	**794**	**1946**	14862	**2138**	**514**
BMP4	HE1	32645	12911	**405**	**99**	15887	**109**	**38**
	HE2	27583	10544	**577**	**141**	16008	**205**	**67**

c

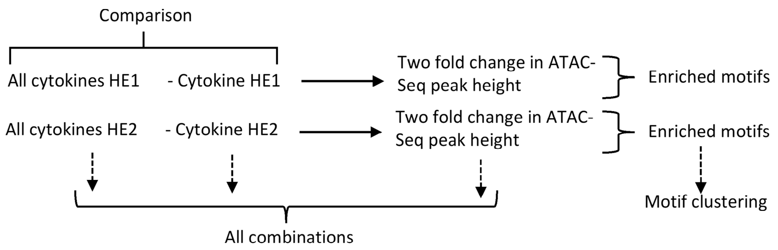

d

e

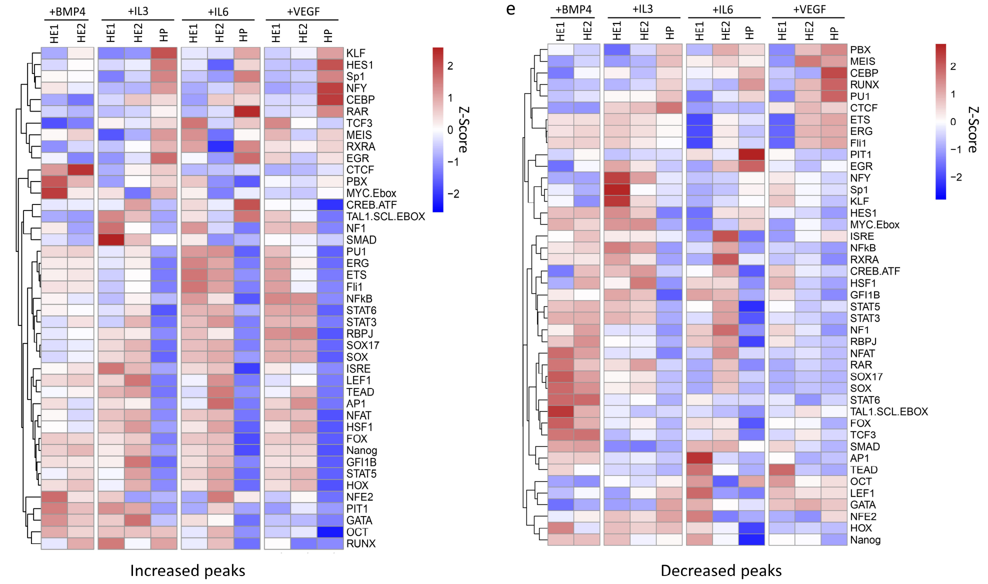

Supplementary Fig. 4: Identification of cytokine responsive enhancer elements. a) Percentage of ATAC peaks from serum free differentiation culture overlapping with ATAC peaks from serum differentiation cultures (n=2). b) number of cytokine-responsive distal and promoter ATAC-peaks containing fragments scoring positive in our assay as compared to the total number of distal and promoter ATAC sites; c-e) Motif enrichment analysis of all distal ATAC peaks irrespective of enhancer activity in the presence and absence of the indicated cytokines, c) overview of motif enrichment strategy, d-e): TF binding motif enrichment in distal ATAC peaks that are increased (d) or decreased (e) in the presence of the indicated cytokines. Z-scores were calculated by rows. Position-weight matrices used for this analysis can be found in Supplementary Table 1 in Supplementary Notes.

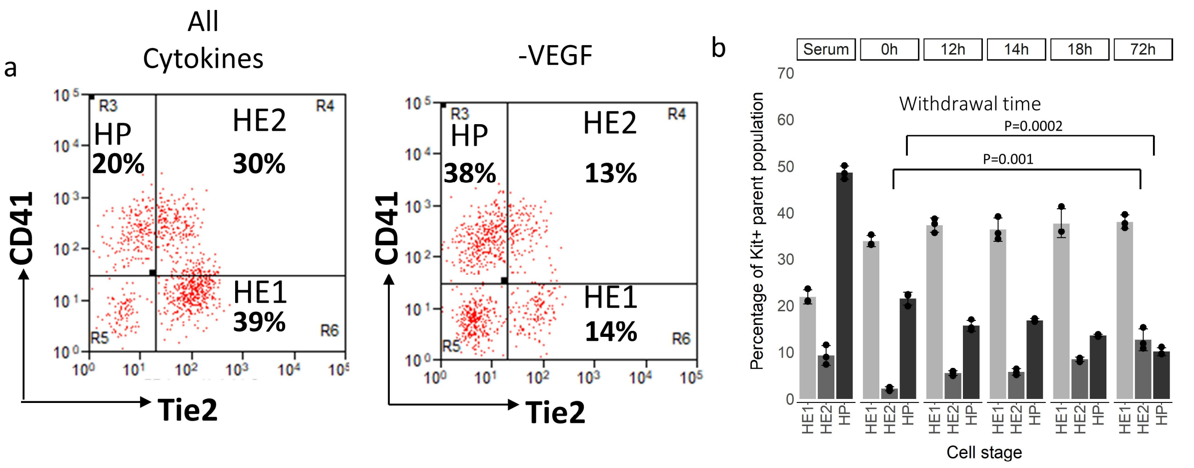

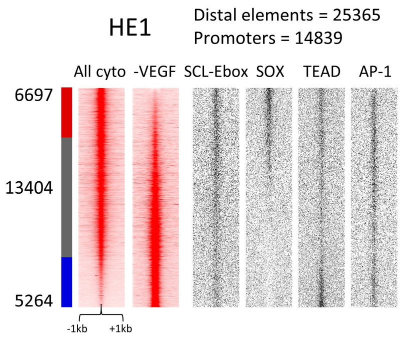

HE1 – Enriched; n = 6697

Motif Sequence	Motif	%sites	%bg
	ETS	69.36	24.47
	SOX	41.39	13.37
	SCL-Ebox	31.33	17.16
	TEAD	29.61	15.23
	FOX	28.24	13.68
	AP-1	16.23	4.15
	ATF-CREB	10.57	3.54
	MEF2	10.17	4.09

HE1 – Depleted; n = 5264

Motif Sequence	Motif	%sites	%bg
	ZFX	60.07	52.09
	SMAD	54.79	43.13
	TEAD	41.70	14.68
	GATA	17.71	8.60
	AP-1	17.50	5.97
	ATF-CREB	14.58	8.83

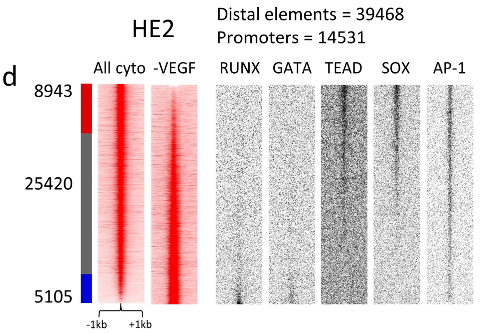

HE2 – Enriched; n = 8943

Motif Sequence	Motif	%sites	%bg
	ETS	62.75	26.64
	TEAD	48.27	27.79
	SOX	36.91	11.36
	SCL-EBox	22.58	13.19
	AP-1	21.45	5.75
	ATF-CREB	13.81	5.39
	MEF2	10.33	5.01

HE2 – Depleted; n = 5105

Motif Sequence	Motif	%sites	%bg
	ETS	46.60	18.18
	SCL-EBox	42.29	32.63
	ATF-CREB	33.14	23.89
	RUNX	23.92	8.59
	GATA	19.69	8.90

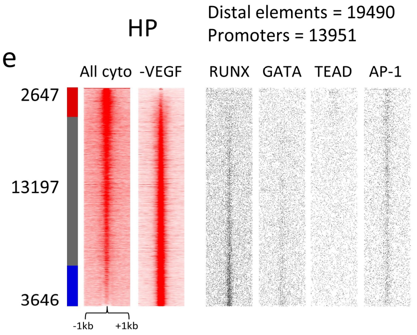

HP – Enriched; n = 2647

Motif Sequence	Motif	%sites	%bg
	ETS	27.02	13.09
	SOX	18.01	11.12
	AP-1	16.81	6.77
	TEAD	12.23	5.63

HP – Depleted; n = 3646

Motif Sequence	Motif	%sites	%bg
	PU.1	44.32	12.25
	RUNX	39.22	2.29
	GATA	17.44	8.79
	AP-1	13.36	6.29

Supplementary Fig. 5: VEGF suppresses blood progenitor development. a) Representative FACS analysis of cKIT+ cells stained with antibodies against Tie2 and CD41 in the presence and absence of VEGF highlighting the percentage of HE1, HE2 and HP cells in the cultures. b) Proportion of HE1, HE2 and HP cells after VEGF was withdrawn at different times during differentiation culture from the beginning of blast culture onwards. Data are represented as mean values +/-SD. Dots showing individual values for n=3 biologically independent experiments. P-values were calculated using two-sided Student's t-test. Source data are provided as a Source Data file. c-e) Comparison of ATAC-Seq profiles of cells grown in medium

with all cytokines (in duplicate) and where VEGF was omitted (-VEGF). The two-fold-difference in peak height was calculated for each peak for each condition using the average of the duplicates and all plots were centered to the peak summit. Peaks were then plotted according to the fold difference to highlight All cytokine and -VEGF specific peaks. The location of individual motifs in the open chromatin regions is plotted alongside. Right panels: Enriched binding motifs for the indicated TFs in the peaks specific for each condition. Motif enrichment analysis in the specific peak populations was performed with HOMER with all enriched motifs displayed passing $P<0.01$ threshold significance (P values calculated using a hypergeometric test in HOMER[3]. Position-weight matrices used for this analysis can be found in Supplementary Table 1 in Supplementary Notes.

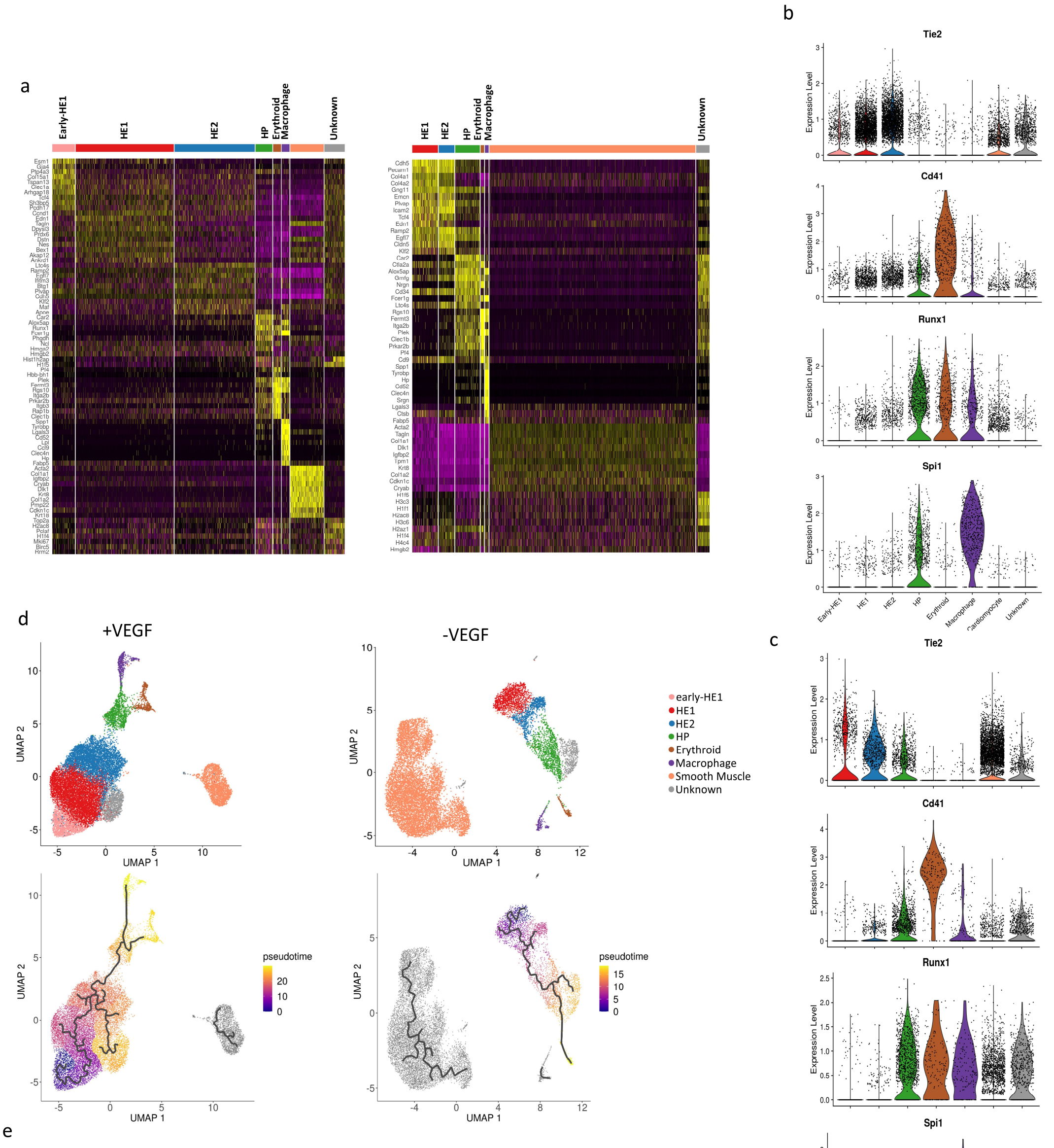

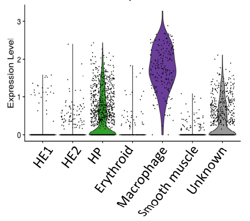

	VEGF Responsive enhancers Total	Linked VEGF Responsive Genes Up	Linked VEGF Responsive Genes Down
HE1 UP	2413	243	94
HE2 UP	2175	145	63
HE1 Down	1354	42	99
HE2 Down	1872	49	63

Supplementary Fig. 6: Single cell RNA-Seq analysis of hemogenic endothelium cultures with and without VEGF. a) Marker gene distribution in the different clusters shown in Fig.7a. b,c) Expression levels of the indicated marker genes in ES-derived immature and mature cell populations from All Cytokine (b) and VEGF omission (c) cultures, demonstrating the purity of the clusters. d) Pseudotime trajectory analysis of cells derived from All Cytokine (d, +VEGF) and VEGF omission cultures (-VEGF) of the clusters depicted in Fig. 7b. e) Number of VEGF responsive genes linked to VEGF-responsive enhancer elements as measured by a 2-fold change in the ATAC-Seq signal. Expression was measured by using aggregated single cell expression values for the purified HE1 and HE2 populations using a threshold of 0.25 Log2 fold change as the currently accepted threshold for sc data.

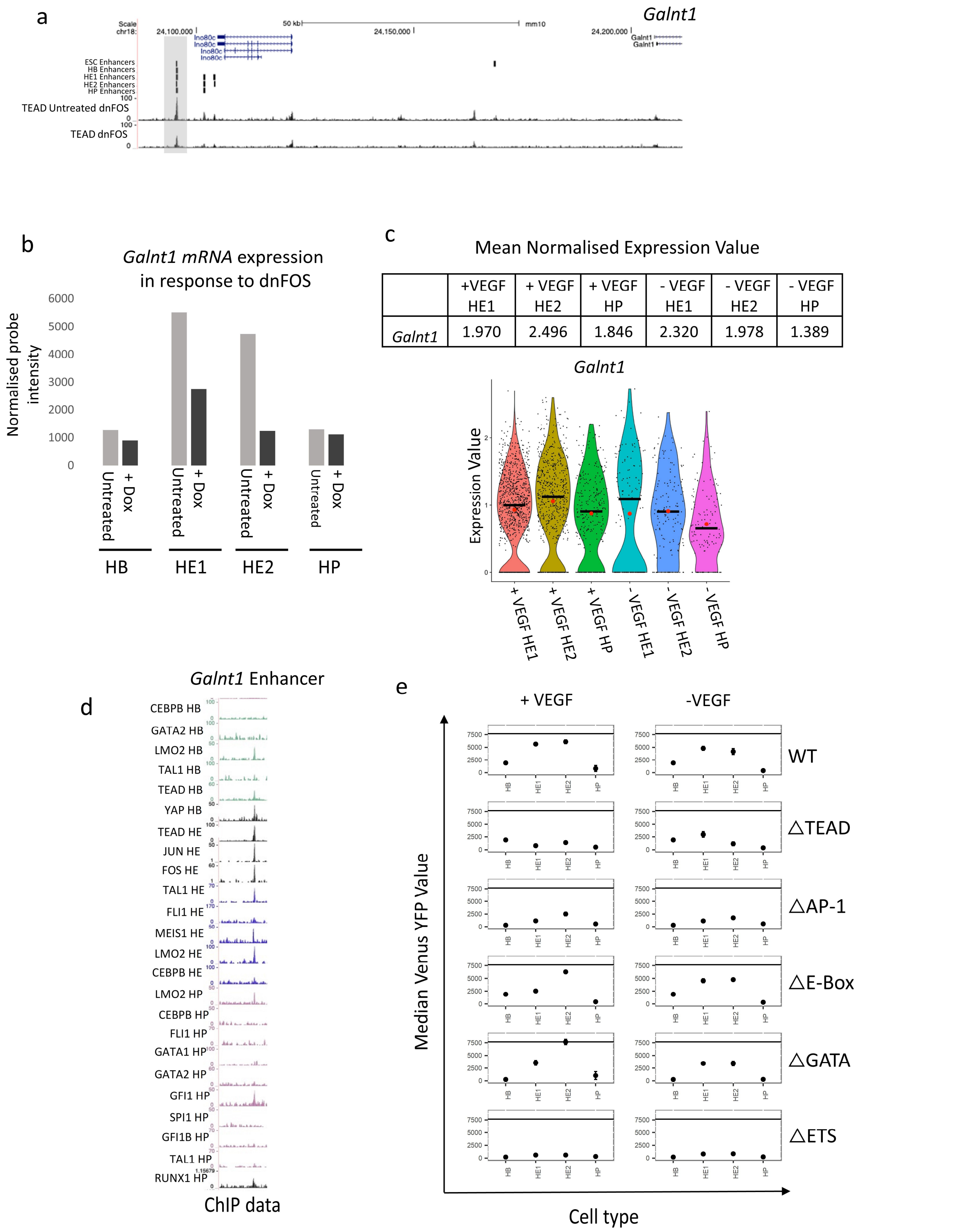

	+VEGF HE1	+ VEGF HE2	+ VEGF HP	- VEGF HE1	- VEGF HE2	- VEGF HP
Galnt1	1.970	2.496	1.846	2.320	1.978	1.389

Supplementary Fig. 7. Interaction between TEAD4 and AP-1 factors at the *Galnt1* enhancer - AP-1 is required for TEAD4 binding and enhancer activity. a) UCSC browser screenshot showing the binding of TEAD4 to the *Galnt1* enhancer with or without expression of a Doxycycline inducible dominant negative FOS (dnFOS) peptide. The *Galnt1* enhancer is highlighted with a grey bar. The *Ino80* gene is constitutively expressed and *Galnt1* was shown to be co-regulated with the presence and absence of a DHS at this enhancer[4]. dnFOS expression resulted in a greater than 2-fold decrease in peak height b) *Galnt1* mRNA

expression with and without dnFOS induction (merged duplicate microarray data[20]). Source data are provided as a Source Data file. c) Mean normalized (Seurat log normalized) *Galnt1* mRNA expression in the HE1 / HE2 populations as measured by scRNA-Seq and violin plots of *Galnt1* mRNA expression value for HE1, HE2 and HP populations cultured with and without VEGF (see below) from 6 clusters for n=2266 cells. Violin plot of expression value with black line representing the median, red dot the mean and black dots the spread of the data. d) Genome Browser screenshot depicting binding of the indicated transcription factors at the different developmental stages to the *Galnt1* enhancer. Note that this enhancer is bound by RUNX1 in the HP which binds to a site overlapping with the TEAD::AP-1 element. Data from Goode et al. [5] and Obier et al.[6] e) Reporter gene activity driven by wild type and mutated *Galnt1* enhancer elements in the presence and absence of the VEGF measured as median fluorescence of all cells. The horizontal line represents the highest baseline measured in the presence of VEGF. Data are presented as mean values +/-SD. n=3 biologically independent experiments. Source data are provided as a Source Data file.

2. Supplementary Notes

Supplementary Notes Table 1: Logos showing position weight matrices used for motif enrichment and motif colocalization analysis

motif	logo	motif	logo	motif	logo
AP-1		ISRE		RARG	
CEBP		KLF		RBPJ	
CREB.ATF		LEF1		REST	
CTCF		MEIS		RUNX	
EGR		MYC.EBOX		RXR	
ERG		NANOG		TCF3	
ESRRA		NF1		SMAD	
ETS		NFAT		SOX	
Fli1		NFE2		SOX17	
FOX		NFκB		Sp1	
GATA		NFY		STAT3	
GFI1b		OCT		STAT5	
HES1		PBX		STAT6	
HOX		PIT1		SCL.TAL1.EBOX	
HSF1		PU.1		TEAD	

Supplementary Notes Table 2: Primers used to add Gateway ® linkers to ATAC-Seq material

Name	Forward	Reverse
**AttB tn5**	GGGGACAAGTTTGTACAAAAAAGCAGGCTTCGTCGGCAGCGTCAGATGTGTATAA<br>GAGACAG	GGGGACCACTTTGTACAAGAAAGCTGGGTGTCTCGTGGGCTCGGAGATGTGTAT<br>AAGAGACAG

Supplementary Notes Table 3: Synthesised enhancer sequences

Name	Sequence
RUNX1-+23-WT	GGGGACAAGTTTGTACAAAAAAGCAGGCTCAGCAAGGAGCGATGGAGGGATGGTGTGAGGAGGAGACAGGAAGGGAGGCGGTGACACAGGCCT<br>TCACAGGGCCGCTGCAGCTAGGAACTGCTGCAAAAGCAAGCTGCCCACGTTATCAGTGGCGCGCAGGCCTGCGGTTTTCTCGCTCTTGCAACCCGGC<br>TTCAACTGCCGGTTTATTTTTCGACAAACAGGATGCCTCCATCTGAGGCTGTCAGCATCCCAGGCTCTTTGAGAAGAAAAGAGGTAGTGAGGGCCCC<br>ACCCTAGAGCCCAGCCGGGAGGGGTGGGTGAGAGGTCCTGTTTCTAGATGCTTCCCAGAGAAGTGAGAACCTAGCAGGTGCAGTGGCCAGGGTTC<br>AGGAGCGTGTCAGGAAACCCAGCTTTCTTGTACAAAGTGGTCCCC
RUNX1-+23-dRUNX1	GGGGACAAGTTTGTACAAAAAAGCAGGCTCAGCAAGGAGCGATGGAGGGATGGTGTGAGGAGGAGACAGGAAGGGAGGCGGTGACACAGGCCT<br>TCACAGGGCCGCTGCAGCTAGGAACTGCTGCAAAAGCAAGCTGCCCACGTTATCAGTGGCGCGCAGGCCTGGCCTTTTCTCGCTCTTGCAACCCGGC<br>TTCAACTGCCGGTTTATTTTTCGACAAACAGGATGCCTCCATCTGAGGCTGTCAGCATCCCAGGCTCTTTGAGAAGAAAAGAGGTAGTGAGGGCCCC<br>ACCCTAGAGCCCAGCCGGGAGGGGTGGGTGAGAGGTCCTGTTTCTAGATGCTTCCCAGAGAAGTGAGAACCTAGCAGGTGCAGTGGCCAGGGTTC<br>AGGAGCGTGTCAGGAAACCCAGCTTTCTTGTACAAAGTGGTCCCC
RUNX1-+23-dTEAD	GGGGACAAGTTTGTACAAAAAAGCAGGCTCAGCAAGGAGCGATGGAGGGATGGTGTGAGGAGGAGACAGGAAGGGAGGCGGTGACACAGGCCT<br>TCACAGGGCCGCTGCAGCTAGGAACTGCTGCAAAAGCAAGCTGCCCACGTTATCAGTGGCGCGCAGGCCTGCGGTTTTCTCGCTCTTGCAACCCGGC<br>TTCAACTGCCGGTTTATTTTTCGACAAACAGGATGCCTCCATCTGAGGCTGTCAGCGTCAAAGGCTCTTTGAGAAGAAAAGAGGTAGTGAGGGCCCC<br>ACCCTAGAGCCCAGCCGGGAGGGGTGGGTGAGAGGTCCTGTTTCTAGATGCTTCCCAGAGAAGTGAGAACCTAGCAGGTGCAGTGGCCAGGGTTC<br>AGGAGCGTGTCAGGAAACCCAGCTTTCTTGTACAAAGTGGTCCCC
RUNX1-+3.7 -WT	GGGGACAAGTTTGTACAAAAAAGCAGGCTAGTTCCTGGGAGTCTCAGTCGAGTCTTATCGTCTTGCTTAGGCAGACTAAGTGGGAACCTGGTGTGG<br>GGCGATAGAGGGGGAAAGGAGCTCCCTGTGGTAGACCACATTCTTTTTGCGTGCCAGTGTCTCAGATGCTGGCCCGACTCTCACGAGAAAGGGAAC<br>AATGGCTTGCTTGGTTCCCCATCTCTGTTTTCTCCCGTTTCCACAGTGGTATCAGGCTGAAAGTGAGTTACAGGTTTCCTCCTGCGAGGATTGCTGGG<br>ACTGATAAAGTACCATCCCAGGAGGAAAACAAGATGCTGAAATTACAGGCAGAAGCAGCCACCAGTGAGGAGCCCTAGGAGTGGCAGGCTGTGGT<br>GTGGGGGGCCAGGAACCCAGCTTTCTTGTACAAAGTGGTCCCC
Galnt1-WT	GGGGACAAGTTTGTACAAAAAAGCAGGCTTGAGTCACCCCGACTCAAGCAGAGCATCGTAAATACATTAAGACACAAGACTGACATGTATATGACA<br>CACACCAGAGCAGCAGCAGAAACATCCTGCCATTTGAACCATGGATTGAGGGCTGCAGGACTGGGAATGACCCAGTGCAGCGTTGGCAAGGATTCA<br>ACACAGCGCATGCGCAGAAGTCTGCAAGTCCTGTAACACTGCGGTCCACGGGGCCCACTAGATTTCACCAGTGGGCGAAAGGATGGAAAGAGCACT<br>TTTCCAGTCTCTTCTTGCGGATCCTCAAGCACTCTGCCTTTCATGCAGGCCAGAGTTCTTGGGGGCTGTGGGTTGCTCCCACATGGAAAGTCCCAAGG<br>CCGTTGCAGGATGTCCTCCACAGAGTCCCATCCTGTCCTTATCACAGCTGCAGGCAGCCTGGGCCACATTCCTGTCTCCTGAGTCACTGGTGTGGCCT<br>GGGCTCCTCGCTCTGGAAACCAGGGCACTGCCTGCTCAGTCTTTGGCTTGTCTTTACTCCTCCATACATACCCAGCTTTCTTGTACAAAGTGGTCCCC
Galnt1-dTEAD	GGGGACAAGTTTGTACAAAAAAGCAGGCTTGAGTCACCCCGACTCAAGCAGAGCATCGTAAATACATTAAGACACAAGACTGACATGTATATGACA<br>CACACCAGAGCAGCAGCAGAAACATCCTGCCATTTGAACCATGGATTGAGGGCTGCAGGACTGGGAtcGACCCAGTGCAGCGTTGGCAAGGATTCA<br>ACACAGCGCATGCGCAGAAGTCTGCAAGTCCTGTAACACTGCGGTCCACGGGGCCCACTAGATTTCACCAGTGGGCGAAAGGATGGAAAGAGCACT<br>TTTCCAGTCTCTTCTTGCGGATCCTCAAGCACTCTGCCTTTCATGCAGGCCAGAGTTCTTGGGGGCTGTGGGTTGCTCCCACATGGAAAGTCCCAAGG<br>CCGTTGCAGGATGTCCTCCACAGAGTCCCATCCTGTCCTTATCACAGCTGCAGGCAGCCTGGGCCACgaTCCTGTCTCCTGAGTCACTGGTGTGGCCT<br>GGGCTCCTCGCTCTGGAAACCAGGGCACTGCCTGCTCAGTCTTTGGCTTGTCTTTACTCCTCCATACATACCCAGCTTTCTTGTACAAAGTGGTCCCC
Galnt1-dAP-1	GGGGACAAGTTTGTACAAAAAAGCAGGCTTGtGcgACCCCGACTCAAGCAGAGCATCGTAAATACATTAAGACACAAGACTGACATGTATATGACAC<br>ACACCAGAGCAGCAGCAGAAACATCCTGCCATTTGAACCATGGATTGAGGGCTGCAGGACTGGGAATGACCCAGTGCAGCGTTGGCAAGGATTCA<br>ACACAGCGCATGCGCAGAAGTCTGCAAGTCCTGTAACACTGCGGTCCACGGGGCCCACTAGATTTCACCAGTGGGCGAAAGGATGGAAAGAGCACT<br>TTTCCAGTCTCTTCTTGCGGATCCTCAAGCACTCTGCCTTTCATGCAGGCCAGAGTTCTTGGGGGCTGTGGGTTGCTCCCACATGGAAAGTCCCAAGG<br>CCGTTGCAGGATGTCCTCCACAGAGTCCCATCCTGTCCTTATCACAGCTGCAGGCAGCCTGGGCCACATTCCTGTCTCCTcgGaCACTGGTGTGGCCTG<br>GGCTCCTCGCTCTGGAAACCAGGGCACTGCCTGCTCAGTCTTTGGCTTGTCTTTACTCCTCCATACATACCCAGCTTTCTTGTACAAAGTGGTCCCC
Galnt1-dE-Box	GGGGACAAGTTTGTACAAAAAAGCAGGCTTGAGTCACCCCGACTCAAGCAGAGCATCGTAAATACATTAAGACACAAGACTGACATGTATATGACA<br>CACACCAGAGCAGCAGCAGAAACATCCTGCgAaTTcAACCATGGATTGAGGGCTGCAGGACTGGGAATGACCCAGTGCAGCGTTGGCAAGGATTCA<br>ACACAGCGCATGCGCAGAAGTCTGCAAGTCCTGTAACACTGCGGTCCACGGGGCCCACTAGATTTCACCAGTGGGCGAAAGGATGGAAAGAGCACT<br>TTTCCAGTCTCTTCTTGCGGATCCTCAAGCACTCTGCCTTTCATGCAGGCCAGAGTTCTTGGGGGCTGTGGGTTGCTCCtACATGGAAAGTCCCAAGG<br>CCGTTGCAGGATGTCCTCCACAGAGTCCCATCCTGTCCTTATCAgAGCTGCAGGCAGCCTGGGCCACATTCCTGTCTCCTGAGTCACTGGTGTGGCCT<br>GGGCTCCTCGCTCTGGAAACCAGGGCACTGCCTGCTCAGTCTTTGGCTTGTCTTTACTCCTCCATACATACCCAGCTTTCTTGTACAAAGTGGTCCCC
Galnt1-dGATA	GGGGACAAGTTTGTACAAAAAAGCAGGCTTGAGTCACCCCGACTCAAGCAGAGCATCGTAAATACATTAAGACACAAGACTGACATGTATATGACA<br>CACACCAGAGCAGCAGCAGAAACATCCTGCCATTTGAACCATGGATTGAGGGCTGCAGGACTGGGAATGACCCAGTGCAGCGTTGGCAAGGATTCA<br>ACACAGCGCATGCGCAGAAGTCTGCAAGTCCTGTAACACTGCGGTCCACGGGGCCCACTAGATTTCACCAGTGGGCGAAAGGATGGAAAGAGCACT<br>TTTCCAGTCTCTTCTTGCGGATCCTCAAGCACTCTGCCTTTCATGCAGGCCAGAGTTCTTGGGGGCTGTGGGTTGCTCCCACATGGAAAGTCCCAAGG<br>CCGTTGCAGGATGTCCTCCACAGAGTCCCATCCTGTCCTTAgCACAGCTGCAGGCAGCCTGGGCCACATTCCTGTCTCCTGAGTCACTGGTGTGGCCT<br>GGGCTCCTCGCTCTGGAAACCAGGGCACTGCCTGCTCAGTCTTTGGCTTGTCTTTACTCCTCCATACATACCCAGCTTTCTTGTACAAAGTGGTCCCC
Galnt1-dETS	GGGGACAAGTTTGTACAAAAAAGCAGGCTTGAGTCACCCCGACTCAAGCAGAGCATCGTAAATACATTAAGACACAAGACTGACATGTATATGACA<br>CACACCAGAGCAGCAGCAGAAACcTCCTGCCATTTGAACCATCGATTGAGGGCTGCAGGACTGtGAATGACCCAGTGCAGCGTTGGCAAAGATTCAA<br>CACAGCGCATGCGCAGAAGTCTGCAAGTCCTGTAACACTGCGGTCCACGGGGCCCACTAGATTTCACCAGTGGGCGAAAaGATcGAAAGAGCACTT<br>CTCCAGTCTCTTCTTGCGGcTCCTCAAGCACTCTGCCTTTCATGCAGGCCAGAGTTCTTGGGGGCTGTGGGTTGCTCCCACATGGAgAGTCCCAAGGC<br>CGTTGCAGGtTGTCCTCCACAGAGTCCCcTCCTGTCCTTATCACAGCTGCAGGCAGCCTGGGCCACATTCgTGTCTCCTGAGTCACTGGTGTGGCCTGG<br>GCTCCTCGCTCTcGAAACCAGGGCACTGCCTGCTCAGTCTTTGGCTTGTCTTTACTCCTCCATACATACCCAGCTTTCTTGTACAAAGTGGTCCCC

Supplementary Notes Table 4: PCR primers used for amplifying target enhancers from the mouse genome and adding Gateway ® sites

Name	Forward	Reverse
**Dlk1**	GGGGACAAGTTTGTACAAAAAAGCAGGCTGCAGTATGGTTCCTGGGAC	GGGGACCACTTTGTACAAGAAAGCTGGGTTGGCCGGATGTGGTTATCTG
**Sparc**	GGGGACAAGTTTGTACAAAAAAGCAGGCTACTTCTCAGTTCTCTGTTG	GGGGACCACTTTGTACAAGAAAGCTGGGTGTTTGAACCCTTTGAACCAC
**Pxn**	GGGGACAAGTTTGTACAAAAAAGCAGGCTAGCAGGAGCCTTGGATCTCC	GGGGACCACTTTGTACAAGAAAGCTGGGTGACAGTGCCTCCCACAAAG

**Hspg2**	GGGGACAAGTTTGTACAAAAAAGCAGGCTGAGCCTCAGTAAGGCCCAG	GGGGACCACTTTGTACAAGAAAGCTGGGTGCAGGAGAGGGAAAACAGAG
**Eif2b3**	GGGGACAAGTTTGTACAAAAAAGCAGGCTACCAAGTGACAGATTTCCTG	GGGGACCACTTTGTACAAGAAAGCTGGGTTGTAGGGGCACCCATATATAC
**Cdh5**	GGGGACAAGTTTGTACAAAAAAGCAGGCTTATGGTACTAAGGGATCGTGG	GGGGACCACTTTGTACAAGAAAGCTGGGTCCATGGGGTGGTTTTGTGTG

Supplemental Discussion of the Methodology

Our high-throughput genome-wide enhancer screening method enables the integration of sequences harbouring open chromatin regions into a safe harbour site, allows tracking of enhancer activities in a differentiating population of cells and to study how enhancer activities change as the cells differentiate and respond to signals. Previous methods using open chromatin region-based libraries did not use single integration sites and only studied one cell type[7]. A paper using of the integration system we employed studied only limited genomic regions[8]. We do however acknowledge certain limitations of the method.

By integrating the reporter cassette into the HPRT locus we ensure that the reporter is in open chromatin throughout differentiation, however, this does of course not recapitulate the topology of the endogenous locus for a particular enhancer. It should be noted, however, that this same limitation applies to most other high throughput enhancer screening approaches including the widely used STARR-Seq techniques[9]. To overcome this limitation, we filter all reporter fragments by the chromatin status of their endogenous locus. We have recovered multiple cis-regulatory elements in our screen which have been studied by gene targeting of endogenous loci by other groups. These include the Tal1 -40kb enhancer[10], the +9.5 Gata2 enhancer[11], the -14kb PU.1 enhancer[12], numerous elements of the Sox2 "super enhancer"[13] and multiple enhancers from the ES cell stage identified by CRISPR screen[14]. However, note that (i) we were able to recover individual elements and validate their predicted activity, (ii) use such elements to identify the transcription factors contributing to their activity, (iii) identify which elements are developmentally regulated and (iv) which ones respond to signalling. Our resource provides ample data to validate other important cis-regulatory elements by mutagenesis and genomic editing.

Another limitation shared with techniques such as STARR-Seq is the proximity of the fragments being tested for enhancer activity to the minimal promoter. Within chromatin, Zuin, et al.[15] have shown that proximity to a promoter has a significant effect on the level of enhancer activity. However, their research looks at enhancer proximity on a kilobase level, far beyond what is possible within reporter assays such as ours or STARR-Seq. We identify elements with the potential to stimulate transcription but cannot measure quantitative contribution of a single element within a single locus. This can only be done by genomic editing. But now we know where to edit.

By performing large transfection and cell culture experiments (500 x 10^6 cells per replicate) and using CRISPR/Cas9 to increase integration efficiency we achieve coverage of up to at least 50% of distal open chromatin regions and even more promoter regions as compared to what we find in the original library. However, the integration efficiency of the reporter cassette into the HPRT locus is still a limiting factor of the overall method and the reason large-scale transfections are required. We believe, however, that the advantages of being able to integrate a reporter and monitor enhancer activation throughout differentiation outweighs the technical challenges in performing the experiments.

Finally, we are also aware of the potential issues around using ATAC fragments as an input to the screen. Due to the nature of TN5 cutting at open chromatin the cloned fragments will mostly be sub fragments of an entire putative enhancer. This may mean that in some cases we miss enhancers either because a fragment is not long enough to cover the required transcription factor motifs for the enhancer to be active or because the fragment does not cover the correct part of the open chromatin site. However, we can mitigate some of these problems by introducing a size selection step. In addition, the data have the nevertheless the potential to be used to dissect activity in individual enhancers by studying the negative and positive fragment sequences. Note that the popular ATAC-STARR-Seq method also makes use of similar input material[16] with potential similar issues.

Supplementary Notes Figures and Figure Legends The model depicted in Figure 8 displays the core signalling and transcription factor network regulating the balance between endothelial and hemogenic fate much of which is based on what is known from the literature in terms of functional requirements for the different factors, including our own data. The following figures represent a summary of the data which we have published before to demonstrate the direct involvement of the different factors in regulating the expression of the genes encoding these factors. In Supplemental Figures 1 - 3 we have pooled ChIP, chromatin and enhancer profile data for the most important genes (*Notch1, Sox17, Runx1* and *Dlk1)* based on the data from Goode et al., 2016 [5] and Obier et al, 2016 [6] and this study. These summaries show in fine detail the developmental regulation of the different genes and the relay of transcription factors binding to them during ES cell differentiation in serum forming the basis of the network.

Figure legend for Supplementary Notes Figure 1 - 3: UCSC browser screenshot of the indicated genes with the top panel showing the major transcripts. The position of ATAC-Seq fragments overlapping with promoters and enhancers scoring positive in our assay is indicated by small vertical bars for the five indicated differentiation stages. ChIP data for HB, HE and HP stages are shown below with a different colour code for each stage. Developmental-stage specific cis-elements are highlighted by boxes or grey bars. Figure S1 also shows the annotation of previously characterized elements described in Nottingham et al., 2007[17]. The binding TEAD and AP-1 (JUN, FOS) is indicated by a horizontal box. The proximal promoter is indicated by a box to the left of enhancer 1.

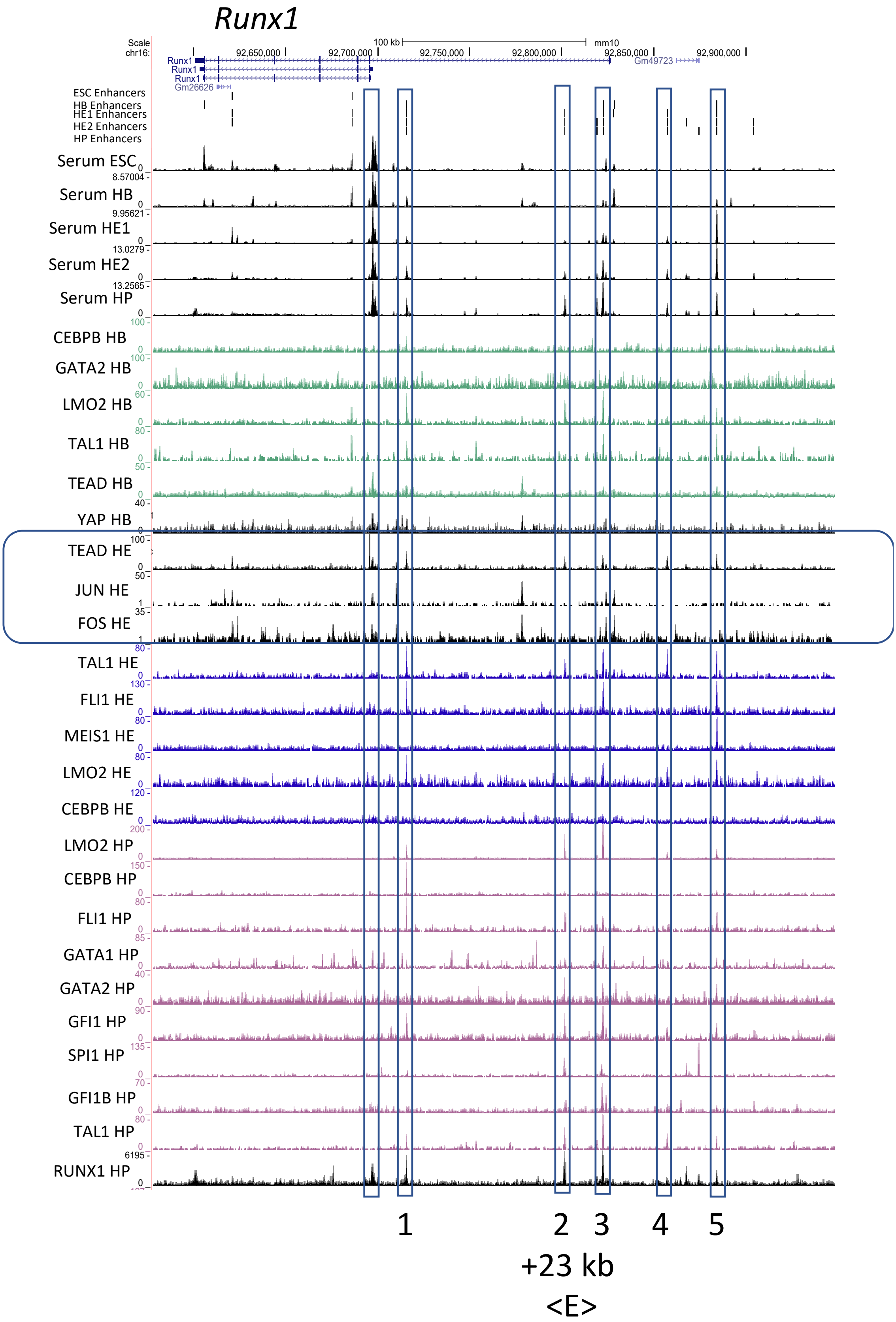

Supplementary Notes Figure 1: Enhancer activity, chromatin structure and developmentally regulated transcription factor binding at the *Runx1* locus.

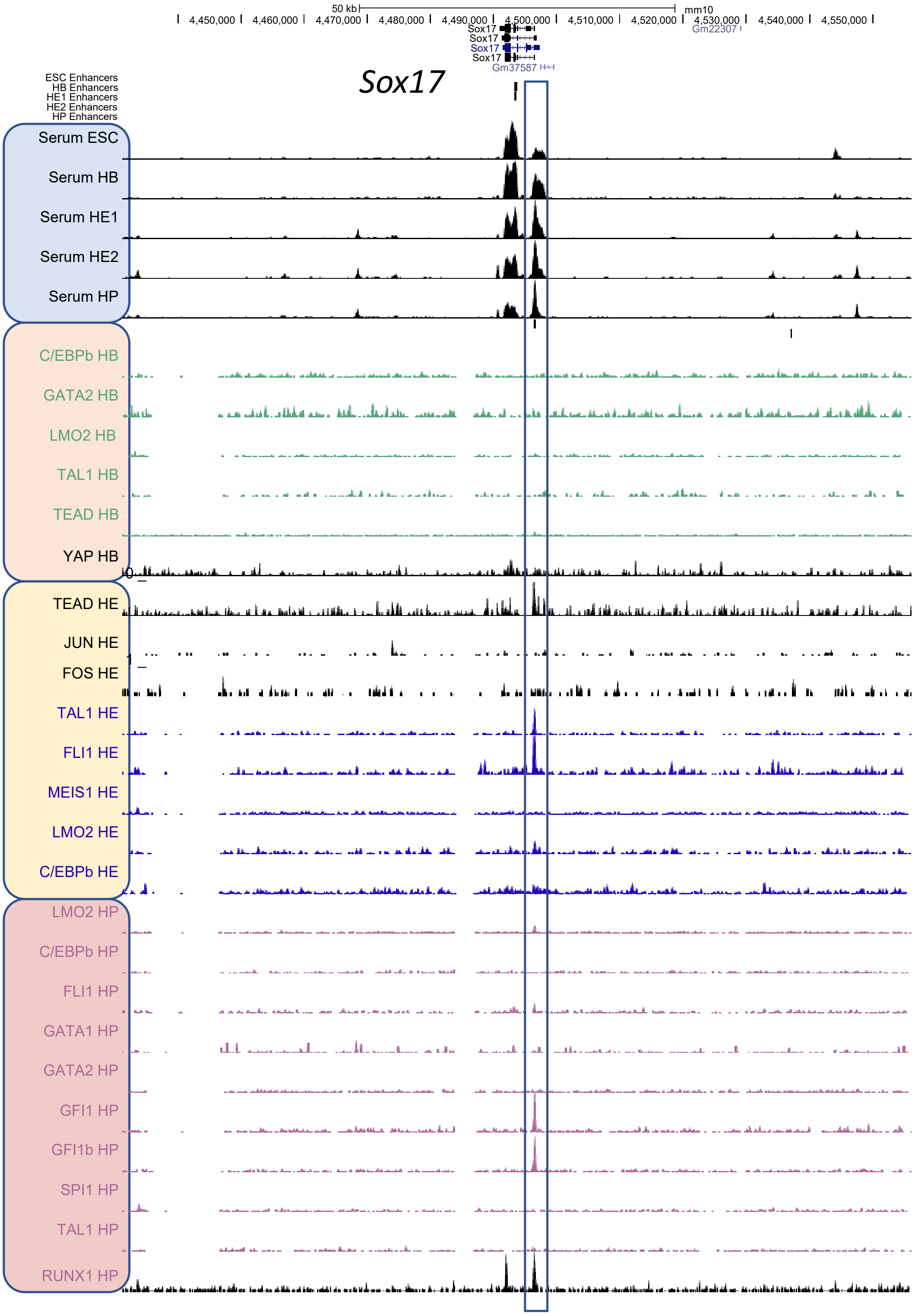

Supplementary Notes Figure 2: Enhancer activity, chromatin structure and developmentally regulated transcription factor binding at the *Sox17* locus.

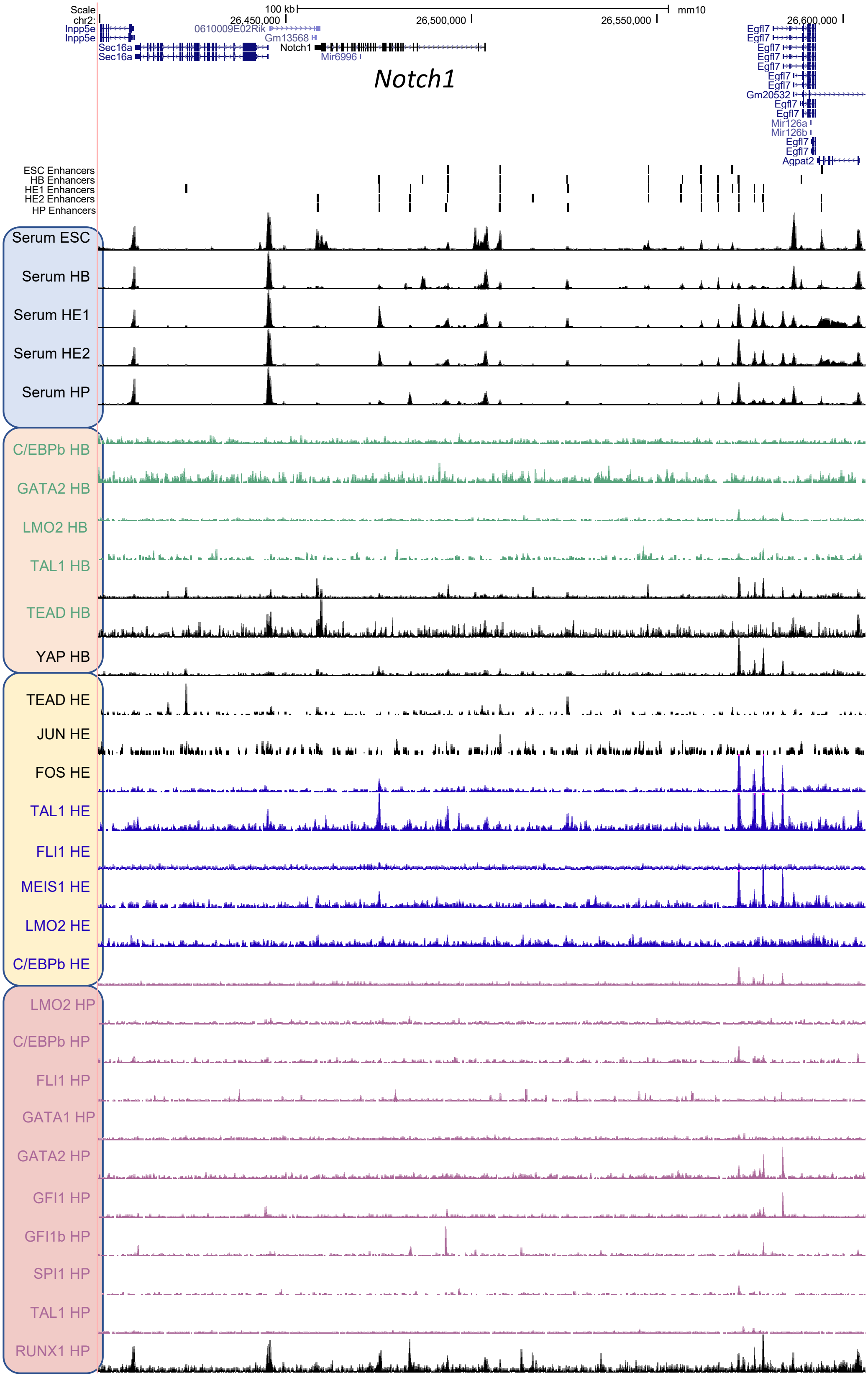

Supplementary Notes Figure 3: Enhancer activity, chromatin structure and developmentally regulated transcription facto binding at the *Notch1* locus.

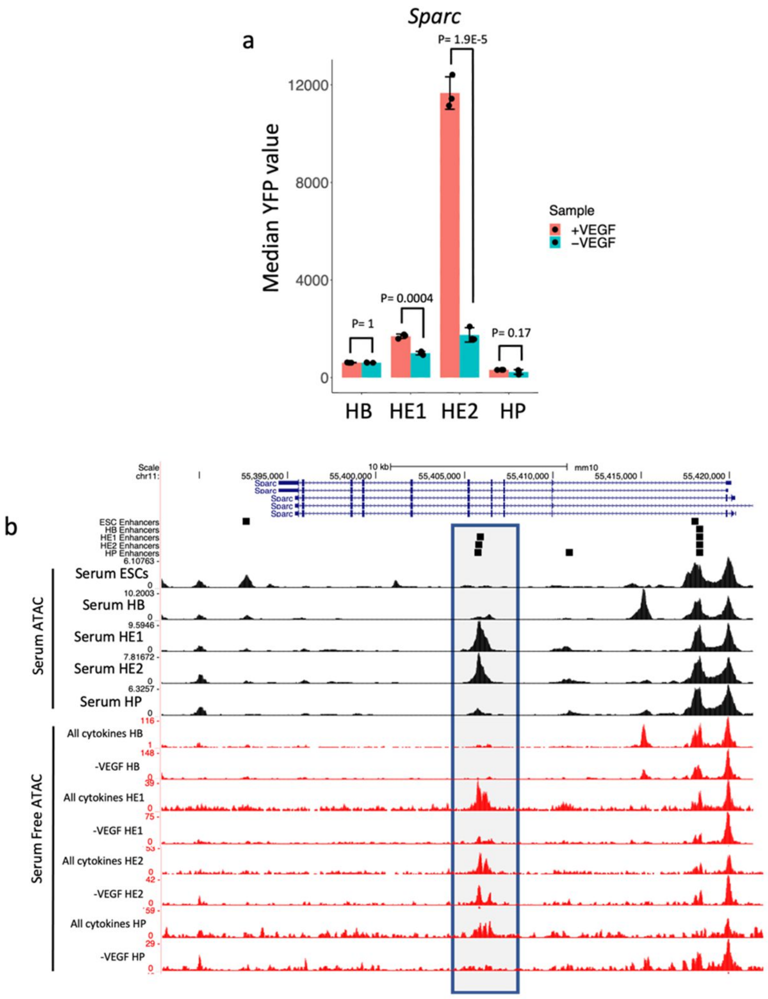

Chr11:55405590-55406032

c

```
ACTTCTCAGTTCTCTGTTGAGCAACTGCAGTGCTGGATTTCTGGATTCAAAGTATTTTCAAGAGTGAC
TTAAAAGATAGAGCTATTGGCATGAATTCAACTGAGCTGTGAGATCCAATGGTGTAGAATCAGGTGTC

           ETS          RUNX      SOX17                ETS

CACAAAGGAACAGGAAGAAGAACCCACAGTAGACAATGGGCCCAGCTCTGACAGGAAACAAAGGCTGG


         FLI1          AP-1                   SOX17

GCATGGAAGAGGAAAGGGATATGACTCAAGACCCCAGGCCCAGCCTTTGTTCCAGCAAGCCCTCCTCA
TTCTCCCCAGACATCCTTAACTGTCCCCTTGAGAGCTCAAGTGGATGCTGGGGATTCAAACTCCGTCT


                                  AP-1                 TAL1

TCATGGTTGTACAGCAAGTGACCTTACCCACTGAGTCATTTCCTTGGGGCCACCATCTGCACCAATGT
CTTGTTAAAAAGCCGTGGTTCAAAGGGTTCAAAC
```

Supplementary Notes Figure 4: The *Sparc* enhancer is VEGF responsive. a) Activity of the *Sparc* enhancer element highlighted in b) in the presence and absence of VEGF at the different developmental stages. Data are presented as mean values +/-SD. Dots showing individual values for n=3 biologically independent experiments. P values calculated using two-sided Student's t-test. b) Presence of absence of the ATAC-Seq site marking the indicated enhancer region at the different developmental stages and cytokine conditions.

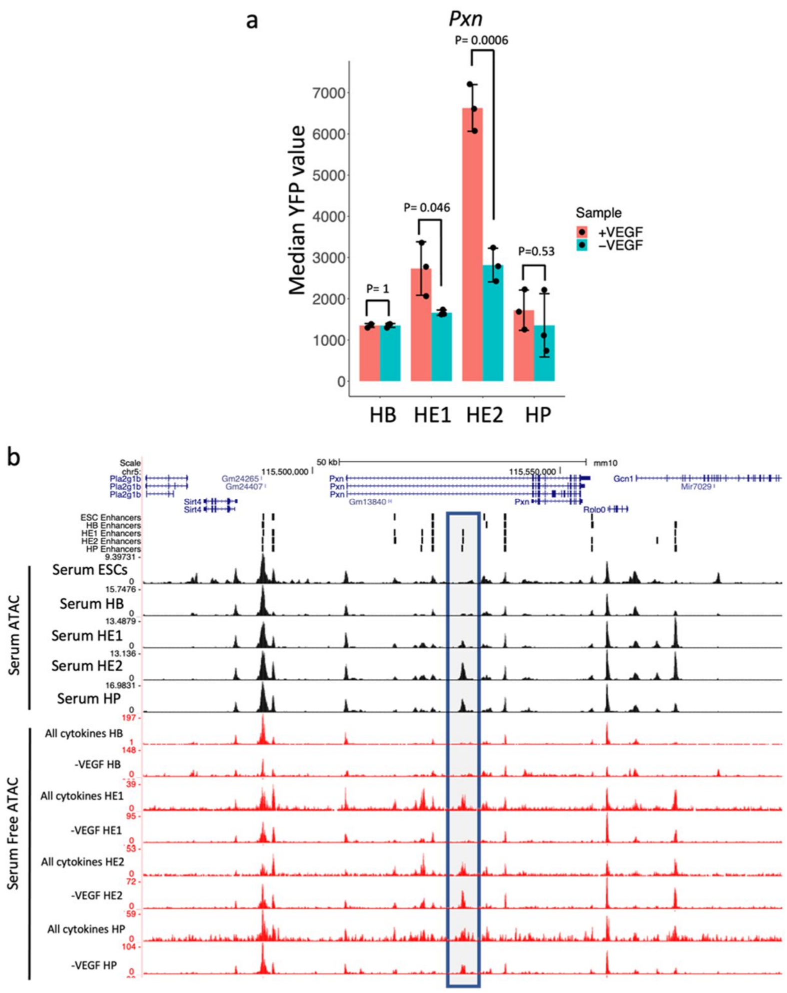

c

Chr5:115530007-115530483

GCAGGAGCCTTGGATCTCCCTCTGCCTCTGAAGCCAGTGGGCAGACTCCACCAGTCAGGGTCCGCGAG
GGCTGGCGTGTGCTTGGCTTGAGGCTCCAGCTCAGGGTCAGTGGGCTTCTTGCAGGCTGGTCATATGA

PU.1/Fli1

CCTTGCTTGTGTTCTG**CTTCCTCC**TCCTGCATCCTACTTCCCTTGCATACGAGCCCTGTCAGCCAGGC

RUNX RUNX

TG**AGCCCACA**GGTCCCCTGTCAGCCAGGCAGAGCCCACAGGTCCCCTGTCAGCCAGGCTG**AGCCCACA**

-RUNX---

--RBPJ--

GGTCCCCAACACATTTTGTTCCAGGAAAGCCTGTTTTCCGTTTTTTCCTG**TGTGGTTTCCCAG**TCTGA

ETS RBPJ

CTTTGCAGCCACCTTGTGCTTCCTCCCTGT**CCAGGAAGA**GGCAGA**TTTCCCACT**GTGAGAGTTGGAGA
AGATCTTCCCTCTAGTGGGACAGGGACAGGCTGGAGCTGTCTGTCCCTCCTTTGTGGGAGGCACTGTC

Supplementary Notes Figure 5: The *Pxn* enhancer is VEGF responsive. a) Activity of the *Pxn* enhancer element highlighted in b) in the presence and absence of VEGF at the different developmental stages. Data are presented as mean values +/-SD. Dots showing individual values for n=3 biologically independent experiments. P values calculated using two-sided Student's t-test. b) Presence of absence of the ATAC-Seq site marking the indicated enhancer region at the different developmental stages and cytokine conditions.

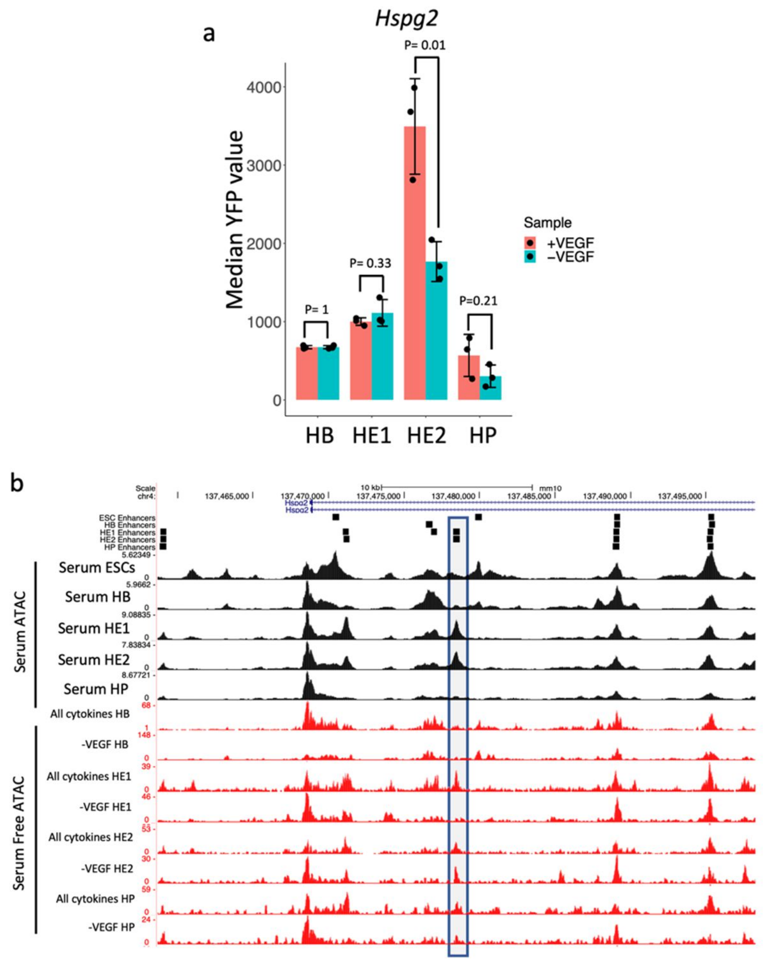

c

```
Chr4:137478251-137478690

 TGAGCCTCAGTAAGGCCCAGCTCAGAAGCCATTCATTCATAGTAAGTTGTTGGGGGACCAGTTCGGC

                         Fli1

TCCTGTTGTCAGGACTGGCTTCCTGGAGGAAGTGGGCCTGAAAATTGGGTGGGTTTGGATATGCTTAG

                                                                 SOX17

GGAACAGGAAGCATCAGGCAGAACTCCCAGCCTCCTCGGGAGGCCCCTCAGCCTGGATCCTATTGTCC

   SOX17          ETS

CCATTGTCTTGGGTGTACAGGAACTGGAAGGAAGAACACTTCGCCTGCTGCTGGAGCTGTTAAAGCTG

             Fli1

AGGGAAGAATGAGAGGAAGTCCCCAAGATAGCTGAGGGGGACATCCACCTTAGCACACCCCCCTCAAC

CACCTAGTCAGACAATTAGGCAAGAAGTCAATGCCTGGTCTCCGCTTGGTCACAGCCACACCTCGTTC

TCTTTGCTGAGCCTCTGTTTTCCCTCTCCTGC
```

Supplementary Notes Figure 6: The *Hspg2* enhancer is VEGF responsive. a) Activity of the *Hspg2* enhancer element highlighted in b) in the presence and absence of VEGF at the different developmental stages. Data are presented as mean values +/-SD. Dots showing individual values for n=3 biologically independent experiments. P values calculated using two-sided Student's t-test. b) Presence of absence of the ATAC-Seq site marking the indicated enhancer region at the different developmental stages and cytokine conditions.

a)

+23 kb *RUNX1* enhancer (2)

CAGCAAGGAGCGATGGAGGGATGGTGTGAGGAGG**AGACAGGAAGG**GAGGCGGTGACACAGGCCTTCAC

GATA **RUNX**

AGGGCCGCTGCAGCTAGGAACTGCTGCAAAAGCAAGCTGCCCAC**GTTATCA**GTGGCGCGCAGGCC**TGCGGTTT**TCTCGC

GCC

TAL1 **TEAD**

TCTTGCAACCCGGCTTCAACTGCCGGTTTATTTTTCGACAAACAGGATGCC**TCCATCTGAG**GCTGTCA**GCAT**C**CCAGG**C

G AA

TCTTTGAGAAGAAAAGAGGTAGTGAGGGCCCCACCCTAGAGCCCAGCCGGGAGGGGTGGGTGAGAGGTCCTGTTTCTAG

E-box (TCF3)

ATGCTTCCCAGAGAAGTGAGAACCTA**GCAGGTG**CAGTGGCCAGGGTTCAGGAGCGTGTCAGGAA

RUNX1 +3.7 Enhancer (3)

b)

chr16:92822182-92822601

RBPJ TAL1------

AGTTCCTGGGAGTCTCAGTCGAGTCTTATCGTCTTGCTTAGGCAGACTAA**GTGGGAA**C**CTG**GTGTGGG

---TEAD--

--GATA RUNX1----RUNX1

GC**GATAGA**GGGGGAAAGGAGCTCCC**TGTGGTAGACCACATTCTTT**TTGCGTGCCAGTGTCTCAGATGC

[illegible]

SOX17

TGGCCCGACTCTCACGAGAAAG**GGAACAATGG**CTTGCTTGGTTCCCCATCTCTGTTTTCTCCCGTTTC

[illegible]

GATA

CACAGTGGTATCAGGCTGAAAGTGAGTTACAGGTTTCCTCCTGCGAGGATTGCTGGGAC**TGATAAAG**T

TEAD

ACCATCCCAGGAGGAAAACAAGATGCTG**AAATTACAGG**CAGAAGCAGCCACCAGTGAGGAGCCCTAGG

AGTGGCAGGCTGTGGTGTGGGGGGCCAGGA

Supplementary Notes Figure 7: Wild type *RUNX1* enhancer sequences and mutant elements. Sequence of the +23 kb enhancer (a) and the +3.7 kb enhancer (b) with indicated

binding motifs. The position and nature of mutations in the +23 element are depicted under the motif.

a)

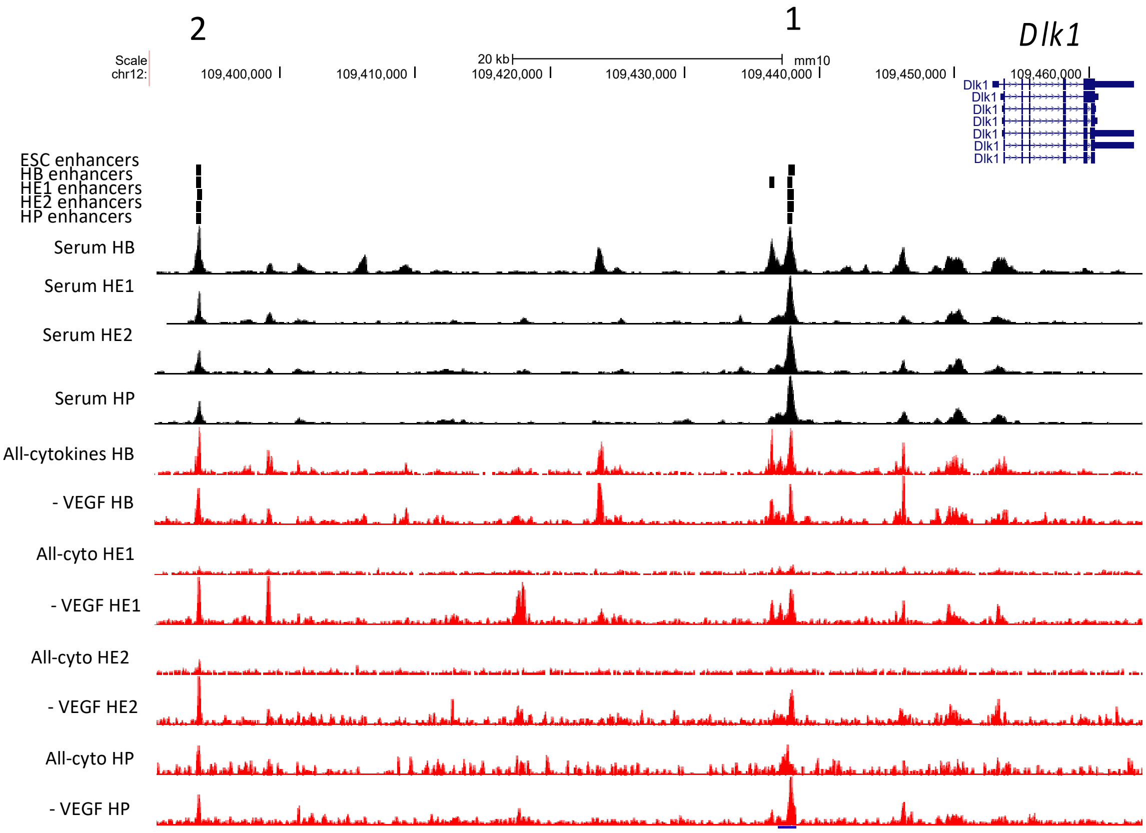

b)

DLK1 Enhancer 1

chr12:109,437,601-109,438,089

Weak RUNX
GCAGTATGGTTCCTGGGACATGAGTTATGACAAGCC**TGTGGCTT**CGTGACTGGAAGGACTGGGGCCCTGGAG
GATA weak IRF
GGAT**TTATCA**CTCTGAAGAGAACCTTCAGAGGGCTTCATTAGGCT**GAAA**A**CGA**T**A**A**T**GAATAGGCTCCCCTTG
GATA---------E box
CCTGCATTCATTCACAACGGCTCTCCT**CTTATCT**GTTCCCTA**CAGG**A**G**CCACTCTCTGAGGAAATGCAGACATC
TEAD ETS
CCGGGGATGAACTGGTAACTGCTGCAGAGTAGGC**GCCTTCCAG**CCCT**CTTCCTCCT**CCACGTTGGCGAGGCGA
E box
GGCCAGGAGACCCGCCGGCAGGGGGCGCAGCCAGCTCGCCGAGAGCAGGGGCGCTAGGCGGGG**CAGGTG**T

GCCCGGGGAGCCAGCGGGACCCAGGGTGGTAGAAAAGTTGAGGCACGCAAAGGGGGAGTAGGGCGAGCTGC
E box-------------GATA RUNX1
CTTCTGGGGCCTTCAGGCCTGCACTGCCTTC**CAGATAACCACA**TCCGGCCATGTAT

Supplementary Notes Figure 8: Chromatin alterations at the *Dlk1* locus indicate the position of VEGF responsive enhancer elements.

a) UCSC browser screenshot of the *Dlk1* locus with the top panel showing the major transcripts. The position of ATAC-Seq fragments overlapping with distal enhancer elements is

indicated by small vertical bars for the five indicated differentiation stages. VEGF responsive enhancer elements are highlighted with a grey shade. b) Sequence of enhancer 1 with indicated binding motifs.

Supplementary Notes References

1. Huber, T.L., Kouskoff, V., Fehling, H.J., Palis, J. & Keller, G. Haemangioblast commitment is initiated in the primitive streak of the mouse embryo. *Nature* **432**, 625-30 (2004).
2. Howell, E.D. *et al.* Efficient hemogenic endothelial cell specification by RUNX1 is dependent on baseline chromatin accessibility of RUNX1-regulated TGFbeta target genes. *Genes Dev* **35**, 1475-1489 (2021).
3. Heinz, S. *et al.* Simple combinations of lineage-determining transcription factors prime cis-regulatory elements required for macrophage and B cell identities. *Mol Cell* **38**, 576-89 (2010).
4. Vijayabaskar, M.S. *et al.* Identification of gene specific cis-regulatory elements during differentiation of mouse embryonic stem cells: An integrative approach using high-throughput datasets. *PLoS Comput Biol* **15**, e1007337 (2019).
5. Goode, D.K. *et al.* Dynamic Gene Regulatory Networks Drive Hematopoietic Specification and Differentiation. *Dev Cell* **36**, 572-87 (2016).
6. Obier, N. *et al.* Cooperative binding of AP-1 and TEAD4 modulates the balance between vascular smooth muscle and hemogenic cell fate. *Development* **143**, 4324-4340 (2016).
7. Murtha, M. *et al.* FIREWACh: high-throughput functional detection of transcriptional regulatory modules in mammalian cells. *Nat Methods* **11**, 559-65 (2014).
8. Dickel, D.E. *et al.* Function-based identification of mammalian enhancers using site-specific integration. *Nat Methods* **11**, 566-71 (2014).
9. Arnold, C.D. *et al.* Genome-wide quantitative enhancer activity maps identified by STARR-seq. *Science* **339**, 1074-7 (2013).
10. Ferreira, R. *et al.* Impaired in vitro erythropoiesis following deletion of the Scl (Tal1) +40 enhancer is largely compensated for in vivo despite a significant reduction in expression. *Mol Cell Biol* **33**, 1254-66 (2013).
11. Dobrzycki, T. *et al.* Deletion of a conserved Gata2 enhancer impairs haemogenic endothelium programming and adult Zebrafish haematopoiesis. *Commun Biol* **3**, 71 (2020).
12. Rosenbauer, F. *et al.* Lymphoid cell growth and transformation are suppressed by a key regulatory element of the gene encoding PU.1. *Nat Genet* **38**, 27-37 (2006).
13. Li, Y. *et al.* CRISPR reveals a distal super-enhancer required for Sox2 expression in mouse embryonic stem cells. *PLoS One* **9**, e114485 (2014).
14. Wang, H.F. *et al.* Defining Essential Enhancers for Pluripotent Stem Cells Using a Features-Oriented CRISPR-Cas9 Screen. *Cell Rep* **33**, 108309 (2020).
15. Zuin, J. *et al.* Nonlinear control of transcription through enhancer-promoter interactions. *Nature* **604**, 571-577 (2022).

16. Wang, X. *et al.* High-resolution genome-wide functional dissection of transcriptional regulatory regions and nucleotides in human. *Nat Commun* **9**, 5380 (2018).
17. Nottingham, W.T. *et al.* Runx1-mediated hematopoietic stem-cell emergence is controlled by a Gata/Ets/SCL-regulated enhancer. *Blood* **110**, 4188-97 (2007).
