# Supplementary material for: A genome-wide relay of signalling-responsive enhancers drives hematopoietic specification
Source: Nat Commun. 2023 Jan 17;14:267. doi: 10.1038/s41467-023-35910-9 (PMC9845378; doi:10.1038/s41467-023-35910-9)
Supplement: Supplementary file 3 — Description of additional Supplementary File [file 41467_2023_35910_MOESM3_ESM.pdf]

<u>Descriptions of additional Supplementary Files</u>

Supplementary Data 1 – Enhancer Positive ATAC sites

Supplementary Data 2 – Enhancer Positive stage specific ATAC sites

Supplementary Data 3 -All Stages Enhancer Positive ATAC site gene associations

Supplementary Data 4 - VEGF Responsive Enhancers linked to VEGF Responsive Genes with GO terms and KEGG pathways

Supplementary Data 5 - List of scRNAseq cell marker genes
