# Supplementary material for: A genome-wide relay of signalling-responsive enhancers drives hematopoietic specification
Source: Nat Commun. 2023 Jan 17;14:267. doi: 10.1038/s41467-023-35910-9 (PMC9845378; doi:10.1038/s41467-023-35910-9)
Supplement: Supplementary file 9 — Reporting Summary [file 41467_2023_35910_MOESM9_ESM.pdf]

nature portfolio

Corresponding author(s): Constanze Bonifer
Benjamin Edginton-White

Last updated by author(s): Dec 11, 2022

Reporting Summary

Nature Portfolio wishes to improve the reproducibility of the work that we publish. This form provides structure for consistency and transparency in reporting. For further information on Nature Portfolio policies, see our Editorial Policies and the Editorial Policy Checklist.

Statistics

For all statistical analyses, confirm that the following items are present in the figure legend, table legend, main text, or Methods section.

n/a	Confirmed	
☐	☒	The exact sample size (*n*) for each experimental group/condition, given as a discrete number and unit of measurement
☐	☒	A statement on whether measurements were taken from distinct samples or whether the same sample was measured repeatedly
☐	☒	The statistical test(s) used AND whether they are one- or two-sided<br>*Only common tests should be described solely by name; describe more complex techniques in the Methods section.*
☐	☒	A description of all covariates tested
☐	☒	A description of any assumptions or corrections, such as tests of normality and adjustment for multiple comparisons
☐	☒	A full description of the statistical parameters including central tendency (e.g. means) or other basic estimates (e.g. regression coefficient) AND variation (e.g. standard deviation) or associated estimates of uncertainty (e.g. confidence intervals)
☐	☒	For null hypothesis testing, the test statistic (e.g. *F*, *t*, *r*) with confidence intervals, effect sizes, degrees of freedom and *P* value noted<br>*Give P values as exact values whenever suitable.*
☒	☐	For Bayesian analysis, information on the choice of priors and Markov chain Monte Carlo settings
☒	☐	For hierarchical and complex designs, identification of the appropriate level for tests and full reporting of outcomes
☒	☐	Estimates of effect sizes (e.g. Cohen's *d*, Pearson's *r*), indicating how they were calculated

Our web collection on statistics for biologists contains articles on many of the points above.

Software and code

Policy information about availability of computer code

Data collection	No data collection software was used in this study
Data analysis	All software used is previously published and open source, details and links below.<br><br>Enhancer Enhancer Sequencing Analysis<br>1) Read trimming – Trim Galore Version 0.6.5 (https://www.bioinformatics.babraham.ac.uk/projects/trim_galore/)<br>2) Alignment to Genome – Bowtie 2.3.5.1 (http://bowtie-bio.sourceforge.net/bowtie2/index.shtml)<br>3) Processing sam/bam files - SAMtools 1.10 (http://www.htslib.org/)<br>4) Processing bed files – BEDTools 2.29.2 (https://bedtools.readthedocs.io/en/latest/)<br>5) Closest gene annotation and motif enrichment – HOMER 4.11 (http://homer.ucsd.edu/homer/)<br><br>ATAC-Seq Analysis<br>1) Sequencing quality checking – FastQC 0.11.9 (https://www.bioinformatics.babraham.ac.uk/projects/fastqc/)<br>2) Read trimming – Trimmomatic 0.39 (http://www.usadellab.org/cms/?page=trimmomatic)<br>3) Alignment to Genome – Bowtie 2.4.4 (http://bowtie-bio.sourceforge.net/bowtie2/index.shtml)<br>4) Peak calling MACS 2.2.7.1 (https://github.com/taoliu/MACS)<br>5) Motif Enrichment – HOMER 4.11 (http://homer.ucsd.edu/homer/)<br>6) Data Visualisation – Java TreeView 1.1.6 (http://jtreeview.sourceforge.net/)<br>7) Data normalisation and processing – R 3.6.1 (Action of the Toes) (https://www.r-project.org/)

Single Cell Analysis
1) Read Alignment – CellRanger 6.0.1 (https://support.10xgenomics.com/single-cell-gene-expression/software/pipelines/latest/release-notes)
2) Further analysis – Seurat 4.0.5 & R 4.1.2 (Bird Hippie) (https://satijalab.org/seurat/ & https://www.r-project.org/)
3) Trajectory analysis – Monocle 3.1.0 (https://github.com/optics-dev/Monocle)
4) Human gene symbol to mouse ortholog conversion - biomaRt package v2.50.0 (https://bioconductor.org/packages/release/bioc/html/biomaRt.html)

For manuscripts utilizing custom algorithms or software that are central to the research but not yet described in published literature, software must be made available to editors and reviewers. We strongly encourage code deposition in a community repository (e.g. GitHub). See the Nature Portfolio guidelines for submitting code & software for further information.

Data

Policy information about availability of data

All manuscripts must include a data availability statement. This statement should provide the following information, where applicable:

- Accession codes, unique identifiers, or web links for publicly available datasets
- A description of any restrictions on data availability
- For clinical datasets or third party data, please ensure that the statement adheres to our policy

All genome-wide data is available from GEO under accession number GSE198775. Previously published data are available under accessions numbers: GSE69101, GSE143460, GSE126496 and GSE79320. A genome browser track hub has also been provided for easy visualization of the data, this can be loaded onto UCSC Genome Browser using the following link: https://genome-trackhub.bham.ac.uk/data/EnhancerHub/hub.txt

Human research participants

Policy information about studies involving human research participants and Sex and Gender in Research.

Reporting on sex and gender	*Use the terms sex (biological attribute) and gender (shaped by social and cultural circumstances) carefully in order to avoid confusing both terms. Indicate if findings apply to only one sex or gender; describe whether sex and gender were considered in study design whether sex and/or gender was determined based on self-reporting or assigned and methods used. Provide in the source data disaggregated sex and gender data where this information has been collected, and consent has been obtained for sharing of individual-level data; provide overall numbers in this Reporting Summary. Please state if this information has not been collected. Report sex- and gender-based analyses where performed, justify reasons for lack of sex- and gender-based analysis.*
Population characteristics	*Describe the covariate-relevant population characteristics of the human research participants (e.g. age, genotypic information, past and current diagnosis and treatment categories). If you filled out the behavioural & social sciences study design questions and have nothing to add here, write "See above."*
Recruitment	*Describe how participants were recruited. Outline any potential self-selection bias or other biases that may be present and how these are likely to impact results.*
Ethics oversight	*Identify the organization(s) that approved the study protocol.*

Note that full information on the approval of the study protocol must also be provided in the manuscript.

Field-specific reporting

Please select the one below that is the best fit for your research. If you are not sure, read the appropriate sections before making your selection.

☒ Life sciences ☐ Behavioural & social sciences ☐ Ecological, evolutionary & environmental sciences

For a reference copy of the document with all sections, see nature.com/documents/nr-reporting-summary-flat.pdf

Life sciences study design

All studies must disclose on these points even when the disclosure is negative.

Sample size	Sample size was not predetermined. A minimum of three independent biological experimental replicates were used to provide statistical significance. Sample sizes for flow cytometry were chosen based on a minimum of 10,000 events after filtering for living cells unless the experimental conditions limited the number of cells obtainable.
Data exclusions	No data were excluded
Replication	All manual experiments were conducted three times, standard deviations were determined, all experiments included within the manuscript were successfully reproduced with no need to discard replicates. Reproducibility of global experiments were determined by correlating repeat data and merging data-sets that were correlated with a correlation coefficient of higher than 0.9 or using only peaks occurring in both

	replicate datasets. All genome-wide data within the manuscript comply to this reproducibility requirement. Genome-wide data from single experiments were only considered if specific features occurred in at least two data-sets.
Randomization	Not relevant for this type of study as all experiments were performed in one cell line.
Blinding	No blinding was performed, all experiments were performed based on standardized protocols and readouts from flow cytometry and genome-wide sequencing are not influenced by the investigator.

Reporting for specific materials, systems and methods

We require information from authors about some types of materials, experimental systems and methods used in many studies. Here, indicate whether each material, system or method listed is relevant to your study. If you are not sure if a list item applies to your research, read the appropriate section before selecting a response.

Materials & experimental systems

n/a	Involved in the study
☐	☒ Antibodies
☐	☒ Eukaryotic cell lines
☒	☐ Palaeontology and archaeology
☒	☐ Animals and other organisms
☒	☐ Clinical data
☒	☐ Dual use research of concern

Methods

n/a	Involved in the study
☒	☐ ChIP-seq
☐	☒ Flow cytometry
☒	☐ MRI-based neuroimaging

Antibodies

Antibodies used	CD309 (Flk1) Biotin - eBioscience, 13-5821-82, Avas12a1<br>CD117 (Kit) APC - bdbioscience, 553356, 2B8<br>CD202b (Tie2) PE - eBioscience, 12-5987-82, TEK4<br>CD41a PE Cyanine7 - eBioscience, 25-0411-82, eBioMWReg30 (MWReg30)
Validation	CD309 (Flk1) Biotin - Manufacturers website shows flow cytometry staining of bEnd-3 cell line with Anti-Mouse CD309 (FLK1) PE using Appropriate isotype controls. 30 publications also cite this antibody https://www.thermofisher.com/antibody/product/CD309-FLK1-Antibody-clone-Avas12a1-Monoclonal/13-5821-82<br>CD117 (Kit) APC - bdbioscience, 553356, 2B8 - Manufacturers website states "Application: Flow cytometry (Routinely Tested)". 15 publications also cite this antibody https://www.bdbiosciences.com/en-us/products/reagents/flow-cytometry-reagents/research-reagents/single-color-antibodies-ruo/apc-rat-anti-mouse-cd117.553356<br>CD202b (Tie2) PE - eBioscience, 12-5987-82, TEK4 - Manufacturers website shows flow cytometry data from bEnd.3 cells stained with 1.0 µg of Rat IgG1 kappa Isotype Control, PE and 1.0 µg of CD202b Monoclonal Antibody, PE. 35 publications also cite this antibody https://www.thermofisher.com/antibody/product/CD202b-TIE2-Antibody-clone-TEK4-Monoclonal/12-5987-82<br>CD41a PE Cyanine7 - eBioscience, 25-0411-82, eBioMWReg30 (MWReg30) - Manufacturers website shows flow cytometry staining of mouse platelets with Anti-Mouse CD61 PE and 0.25 µg of Anti-Mouse CD41 PE-Cyanine7. 42 publications also cite this antibody https://www.thermofisher.com/antibody/product/CD41a-Antibody-clone-eBioMWReg30-MWReg30-Monoclonal/25-0411-82

Eukaryotic cell lines

Policy information about cell lines and Sex and Gender in Research

Cell line source(s)	HM-1 cell lines (no longer commercially available) were genetically modified as described in Wilkinson et al., and provided by the Gottgens lab .
Authentication	DNA from HM-1 cells was used in NGS approaches (ATAC-SEQ) and such data were integrated with other ESC data-sets thus confirming that they were ESCs.
Mycoplasma contamination	We test regularly for Mycoplasma contamination and all cells used tested negative.
Commonly misidentified lines (See ICLAC register)	No such cell lines were used

Flow Cytometry

Plots

Confirm that:

- [x] The axis labels state the marker and fluorochrome used (e.g. CD4-FITC).
- [x] The axis scales are clearly visible. Include numbers along axes only for bottom left plot of group (a 'group' is an analysis of identical markers).
- [x] All plots are contour plots with outliers or pseudocolor plots.
- [x] A numerical value for number of cells or percentage (with statistics) is provided.

Methodology

Sample preparation

Cells were incubated in MACS Buffer (PBS with 0.5% BSA and 2mM EDTA) with florophore labeled antibodies for 15 minutes and then washed twice in MACS and pellted by centifugation at 300 xg for 5 minutes. Cells were resuspended in MACS buffer prior to flow cytometry.

Instrument

MoFLow XDP, CyAn ADP Flow Cytometer (Beckman), CytoFLEX (Beckman)

Software

Summit 5, Cytexpert

Cell population abundance

Purity of samples was determined by running the sorted cells on the CyAN ADP flow cytometer following sorting providing enough cells were available, all tested populations had a purity of > 95%. For enhancer screening experiments cells were combined from several differentiations to obtain enough material.

Enhancer Screen - Replicate 1 No. of Cells
ES-Negative 20,000,000
ES-Low 10,100,000
ES-Medium 2,150,000
ES-High 121,000
HB-Negative 10,700,000
HB-Low 10,130,000
HB-Medium 1,380,000
HB-High 174,000
HE1-Negative 236,000
HE1-Low 96,800
HE1-Medium 62,850
HE1-High 19,139
HE2-Negative 806,000
HE2-Low 320,000
HE2-Medium 164,400
HE2-High 30,800
HP-Negative 835,000
HP-Low 386,000
HP-Medium 189,700
HP-High 28,607

Enhancer Screen - Replicate 2 No. of Cells
ES-Negative 10,000,000
ES-Low 4,900,000
ES-Medium 1,530,000
ES-High 296,000
HB-Negative 10,000,000
HB-Low 4,900,000
HB-Medium 999,000
HB-High 221,400
HE1-Negative 1,055,000
HE1-Low 235,800
HE1-Medium 35,800
HE1-High 15,100
HE2-Negative 1,088,000
HE2-Low 215,000
HE2-Medium 36,000
HE2-High 11,800
HP-Negative 1,147,000
HP-Low 134,000
HP-Medium 16,300

	HP-High 8,200
Gating strategy	Debris was removed by gating the dominant cell population using FSC/SCC and duplets were removed by by gating on the main population for pulse width. All other gates were set based on isotype stained or untransfected control cells. Full gating strategies are in figures S1 and S5.

☒ Tick this box to confirm that a figure exemplifying the gating strategy is provided in the Supplementary Information.
